# The dual burden of obesity: decoding metabolism and female reproductive endocrinology

**DOI:** 10.3389/fphys.2025.1627607

**Published:** 2025-11-14

**Authors:** Yan Chen, Rongyu Wang, Nannan Zhang, Liangzhi Xu

**Affiliations:** 1 Reproductive Endocrinology and Regulation Laboratory, West China Second University Hospital, Sichuan University, Chengdu, China; 2 Department of Obstetrics and Gynecology, West China Second University Hospital, Sichuan University, Chengdu, China; 3 Key Laboratory of Birth Defects and Related Diseases of Women and Children (Sichuan University), Ministry of Education, Chengdu, China; 4 Department of Traditional Chinese Medicine, West China Second University Hospital, Sichuan University, Chengdu, China; 5 National Center for Birth Defect Monitoring, West China Second University Hospital, Sichuan University, Chengdu, China

**Keywords:** obesity, evolution, gut microbiota, insulin resistance, reproductive endocrine dysfunction, polycystic ovary syndrome

## Abstract

The global prevalence of obesity continues to rise, posing a threat to health, especially among women, where obesity can lead to reproductive endocrine disorders. Adipose tissue interacts with endocrine hormones, including insulin, leptin, and sex hormones, resulting in functional abnormalities of the female hypothalamic-pituitary-ovarian axis through various central and peripheral mechanisms. At the same time, systemic inflammation, intestinal microbiota, and metabolites are also implicated in these processes, further linking metabolic imbalance to reproductive endocrine dysfunction. Therefore, targeting these co-regulatory mechanisms is expected to improve metabolic disorders and reproductive endocrine dysfunction in obese women. Strategies for treating obesity include dietary and behavioral interventions, medication, surgical treatment, and traditional and alternative medical therapies, showing benefits for improving reproductive endocrine dysfunction. This review calls on clinicians to pay attention to the impact of obesity on reproductive health in women and proposes possible intervention measures.

## 1 Introduction

Obesity is defined as a body mass index (BMI) exceeding 30 kg/m^2^, while overweight is classified as a BMI between 25 and 29.9 kg/m^2^, as per the World Health Organization (WHO). However, these thresholds may vary for different racial and regional groups. For example, for the Chinese population, overweight is defined as a BMI over 24, and obesity as a BMI over 28, whereas in Asia, overweight is classified as a BMI of 23–24.9, and obesity as a BMI of 25 or above (Elmaleh-Sachs et al., 2023; Zhou and Coorperative Meta-Analysis Group Of China Obesity Task Force, 2002). Over the past few decades, the rate of obesity has increased alarmingly, posing a significant threat to global public health. This is evident in the prevalence, incidence, and economic burden of various major chronic diseases. Obesity contributes to at least 5% of global deaths and is a major risk factor for numerous diseases, including type 2 diabetes mellitus (T2DM), hypertension, cardiovascular disease (CVD), chronic obstructive pulmonary disease (COPD), and cancer. Collectively, these conditions reduce life expectancy (Liu et al., 2024; Loos and Yeo, 2022). According to the latest data from the WHO in 2022, approximately 2.5 billion adults aged 18 and above were classified as overweight, with over 890 million classified as obese. This represents 43% of adults (43% of men and 44% of women) being overweight, and 16% categorized as obese (WHO, 2025). Furthermore, the obesity epidemic extends beyond adults, with over 390 million children and adolescents aged 5 to 19 being overweight, and 160 million being classified as obese (WHO, 2025). Projections suggest that by 2030, the prevalence of obesity will increase by an additional 10%, exacerbating the already significant burden on global public health (Ampofo and Boateng, 2020), highlighting the urgent need for effective public health interventions.

In recent years, there has been an increasing awareness of the impact of obesity on reproductive health, particularly concerning reproductive endocrine functions. Reproductive endocrinology is concerned with the hormonal regulation of reproductive processes and the diagnosis and treatment of disorders related to reproductive hormones in both men and women. Obesity-related issues in male reproductive health include a high risk of developing hypogonadism, impaired spermatogenesis, and erectile dysfunction (Molina-Vega et al., 2018). Obesity in women leads to complex interactions between adipose tissue, insulin, leptin, sex hormones, and other endocrine hormones, which can cause functional abnormalities within the hypothalamic-pituitary-ovarian (HPO) axis. The dysregulation of the HPO axis often initially manifests as menstrual disorders, such as irregular menstrual cycles, abnormal uterine bleeding (AUB), and amenorrhea. If these symptoms are left untreated, they can progress to ovulatory dysfunction and infertility (Mikhael et al., 2019). Despite menstrual irregularities and infertility, obesity in women is also significantly associated with other reproductive endocrine disorders, such as precocious puberty (PP) and polycystic ovary syndrome (PCOS), both of which originate from dysfunction of the HPO axis (Santoro et al., 2004; Biro et al., 2010; Teede et al., 2021). Additionally, systemic chronic inflammation, intestinal microbiota, and metabolites are implicated in these processes, further linking metabolic imbalance to reproductive endocrine dysfunction. In this review, we will explore in detail the correlation and underlying mechanisms between obesity-related metabolic disorders and female reproductive endocrine dysfunction. Furthermore, we will summarize and discuss treatment strategies designed to address these obesity-related reproductive health issues, based on the identified mechanisms.

## 2 Obesity and reproductive endocrine: insights from genetics and evolution

### 2.1 Etiology of obesity

The etiology of obesity encompasses both unmodifiable and modifiable factors. Specific mutations in genes that participate in the leptin-melanocortin pathway, such as those encoding leptin, leptin receptor (LepR), melanocortin-4 receptor (MC4R), and pro-opiomelanocortin (POMC), are recognized to cause monogenic forms of obesity (Butler, 2016). However, the majority of obesity cases are polygenic, involving multiple genetic factors that regulate BMI, energy homeostasis, lipid metabolism, and feeding behaviors. These factors are often implicated in neurodevelopment, indicating that obesity could stem from neurodevelopmental abnormalities (Locke et al., 2015). A recent large-scale study using data from 338,645 individuals in the UK Biobank found that adherence to a healthy lifestyle significantly reduces the risk of obesity and related morbidities (ORM), emphasizing the importance of modifiable factors in obesity’s etiology (Kim MS. et al., 2024).

Epigenetic modifications also play a crucial role in obesity. Environmental factors, including diet and physical exercise, can alter gene expression without changing the DNA sequence, a process known as epigenetic regulation. In cases of obesity, significant methylation changes have been observed in genes associated with energy balance, lipid metabolism, and inflammatory processes within adipose tissue and blood cells. For example, elevated methylation levels of the Pparg, which expresses the peroxisome proliferator-activated receptor gamma (PPARγ) and Lep, which expresses leptin, are found in obese individuals, influencing adipocyte differentiation and satiety regulation (Uddandrao et al., 2024). In mice, high-fat diets (HFD) disrupt hypothalamic histone modifications and DNA methylation, affecting chromatin accessibility in hypothalamic neuroendocrine cells (Ma et al., 2024). Moreover, long non-coding RNAs (lnc RNAs) such as Mist, lincIRS2, lncRNA-p5549, H19, GAS5, and SNHG9 are downregulated, while lncRNA-HOTAIR, involved in adipocyte differentiation, is upregulated in adipose tissue of obese individuals (Erdos et al., 2022; Ghafouri-Fard and Taheri, 2021).

The fat mass and obesity-associated protein (FTO) gene, an m6A demethylase (Jia et al., 2011), modulates lipid synthesis by influencing the expression of genes such as C/EBPα (CCAAT/enhancer-binding protein alpha), PPARγ, and sterol regulatory element-binding protein-1 (SREBP1), which are involved in triglyceride and cholesterol synthesis (Frayling et al., 2007). Recent research has highlighted that epigenetically mature genomic regions in the arcuate nucleus (ARC) of the mouse hypothalamus overlap with genomic regions associated with BMI in humans, suggesting that the epigenetic development of this brain region may influence the risk of obesity (MacKay et al., 2022). Furthermore, studies also show that diet-induced obesity alters the methylation patterns of genes involved in glycolipid metabolism, such as Lep, Ppar-α, and Mgat1, in oocytes and liver cells of both F1 and F2 offspring in rodents (Chao et al., 2024). This suggests that epigenetic modifications induced by environmental or lifestyle factors, such as DNA methylation, can be inherited across generations, potentially affecting the risk of obesity in offspring (King and Skinner, 2020; Takahashi et al., 2023).

Additionally, obesity-related genes, including FTO and Lep, are found to affect both lipid metabolism and reproductive function through shared neuroendocrine pathways in the hypothalamus. These genes link energy status with reproduction by modulating metabolic and reproductive hormones (Salum et al., 2025). This dual regulation highlights how metabolic imbalances contributing to obesity can disrupt reproductive health through genetic and epigenetic mechanisms, underscoring the close link between obesity and reproductive health.

### 2.2 The evolutionary perspective on metabolism and reproduction

The connection between metabolism and reproduction has long been recognized in evolutionary biology. Early research, notably by Charles Darwin and later by Rose Frisch, suggested that body fat (BF) and nutritional status are critical determinants of female fertility. Frisch’s work highlighted that low BF (below 17%) can lead to infertility and delayed puberty, while a minimum of 22% BF is required for normal ovulatory cycles and reproductive health (Frisch, 2000). Subsequent research has demonstrated that an excess of energy and the resulting obesity can impair Hypothalamic-Pituitary-Gonadal (HPG) axis function and reduce fertility in both sexes (Uddandrao et al., 2024), suggesting that reproductive dysfunction may occur when BF or body weight (BW) surpasses a certain threshold.

From an evolutionary perspective, humans have evolved to store fat as a survival mechanism during periods of food scarcity, utilizing it as an energy reserve to ensure survival and reproductive success. However, in contemporary environments characterized by an abundance of high-calorie foods and limited physical activity, this natural fat storage mechanism has become dysregulated, contributing to an increase in obesity prevalence (Speakman, 2013).

John R. Speakman and Joel K. Elmquist’s Dual-Intervention Point (DIP) hypothesis suggests that fat regulation occurs at two points: the Lower Intervention Point (LIP) during scarcity to prevent anorexia and support reproduction, and the Upper Intervention Point (UIP) during abundance to avoid excessive fat accumulation (Speakman and Elmquist, 2022). Over time, evolutionary changes and mutations in the UIP regulation system have led to difficulties in managing obesity in modern environments (Speakman and Elmquist, 2022). “Thrifty genes”, including those linked to leptin, play a crucial role in obesity and metabolic diseases, with mutations leading to leptin resistance (LR) and obesity. Fat also serves as an immune-regulatory organ, producing immune modulators like inflammatory factors like tumor necrosis factor (TNF) and interleukins (ILs), aiding survival in resource-limited environments (Speakman and Elmquist, 2022; Rubio-Ruiz et al., 2015). In modern, resource-abundant and relatively safe environments, the protective role of inflammation has diminished, allowing obesity to contribute to systemic diseases and age-related disorders, including reproductive dysfunction.

### 2.3 Hormonal regulation of metabolism and reproduction

Metabolic hormones, including leptin, insulin, and adiponectin, play a crucial role in female sexual development and reproductive function. Leptin, a hormone secreted by adipose tissue that regulates feeding, signals the brain that sufficient energy reserves are available to support reproduction, acting as a critical factor in the initiation of puberty (de Medeiros et al., 2021). Insulin and insulin-like growth factor 1 (IGF-1) signaling are also involved in regulating reproductive function. For example, in *Caenorhabditis elegans*, food availability activates the insulin/IGF-1 signaling pathway, which promotes growth and reproduction (Murphy and Hu, 2013). In female mice, targeted ablation of neurons that encode the IGF-1 receptor gene disrupts the LH peak, impairing ovulation. Similarly, IGF-1 receptor knockout in hypothalamic kisspeptin neurons results in reduced appetite, BW, and delayed puberty (Murphy and Hu, 2013; Wang M. et al., 2024), underscoring the role of adipose energy reserves (signaled by leptin) and nutrient availability (mediated by IGF-1) in regulating reproductive capacity across species.

Beyond leptin and insulin, various steroid hormones are integral to the regulation of female metabolic and reproductive endocrine functions. These hormones influence both central and peripheral organs involved in foraging and energy metabolism and exert direct effects on the HPO axis. For instance, excess androgen can lead to abdominal obesity and insulin resistance(IR) in females, disrupting normal follicular development and ovulation, thereby contributing to reproductive endocrine disorders such as PCOS (Azziz et al., 2006). Other metabolism-regulating peptides, such as Glucagon-like peptide-1 (GLP-1), irisin, adiponectin, ghrelin, and growth hormone, also regulate feeding and reproductive behaviors through both central and peripheral pathways. These hormones have extensive targets, affecting the brain, especially the hypothalamus, as well as peripheral metabolic organs and gonads, and thus play a crucial role in reproductive endocrine regulation. Disruptions in these hormones, particularly in obesity, lead to dysfunction in the female HPO axis, causing menstrual irregularities and infertility (Khan et al., 2022; Luo et al., 2020; Luo et al., 2021). We now summarize the common targets and mechanisms of these steroid and peptide hormones affecting obesity and reproduction in Table 1 for reference.

**TABLE 1:** Co-regulators of reproduction and metabolism.

Molecules name	Mainly generating site	Signalling pathways	Regulatory targets and effects		References
			Metabolize reproductive endocrine		
Leptin	Adipose tissue	JAK-ATAT PI3k-AKT MAPK CAMP	Central: H, Neuron of AgRP/NPY, POMC, NOS1 Effects ↓appetite and ↑energy consumption	Central: H, Neuron of NPY, GnRH anterior lobe of P. Effects ↑GnRH, LH and FSH.	Liu et al. (2023b), Park and Ahima (2015)
			Peripheral: muscle, liver, adipose, tissue, immune cells and endothelial cells Effects ↑insulin sensitivity, ↓inflammation Regulate adipocyte secretion and endothelial function	Peripheral: ovarian granulosa cells, ollicular membrane cells and oocytes Effects Regulate the endocrine function, follicular development and selection of dominant follicles of granulosa cells	de Medeiros et al. (2021), Pérez-Pérez et al. (2020), Wołodko et al. (2021)
Insulin	pancreas	PI3K/AKT MAPK/EKR	Central: H, Neuron of AgRP/NPY, POMC, and glial cells Effects Regulating food intake and energy expenditure, as well as the homeostasis of fat and glucose metabolism	Central: Kisspeptin neuron, GnRH Neuron, astrocytes Effects Regulating the GnRH release	Brüning et al. (2000), Brothers et al. (2010), Manaserh et al. (2019), Saltiel (2021)
			Peripheral: fat, muscle, liver cells Effects Enhances glucose uptake, promotes hepatic glycogen synthesis, inhibits glycogenolysis and gluconeogenesis, stimulates adipocyte glucose and lipid uptake, fosters fat synthesis, and promotes muscle amino acid uptake for protein synthesis	Peripheral: oocytes, granulosa cells and follicular membrane cells Effects The interaction with LH promotes the generation of androgen in follicular membrane cells, promoting follicle activation growth, and ovulation, and regulating granulosa cell endocrine function	Acevedo et al. (2007), Wu et al. (2014)
Adiponectin	Adipose tissue	LKB1/AMPK PI3K/AKT	Central Neuron of NPY and POMC Effects Regulate appetite and energy consumption	Central: hypothalamus kisspeptin neuron, GnRH neuron Effects ↓ kisspeptin expression, GnRH release and LH production	Rak et al. (2017), Wang and Cheng (2018)
			Peripheral fat, liver, muscle, vascular endothelium, immune system, etc Effects ↑insulin sensitivity, fat ecomposition ↓fat synthesis, inflammation protecting vascular endothelium, etc.	Peripheral follicular membrane cells, granulosa cells, oocytes and luteum Effects Regulating oocyte to reduce division, follicle development and sex hormone synthesis, affect ovarian reserve function	Chabrolle et al. (2007), Cheng et al. (2016), Lagaly et al. (2008), Straub and Scherer (2019)
Gastrin	P/D1 cells in Gastric fundus Islet ε cells	Ca^2+^-CAMK-AMPK-CPT1- UCP2-MTOR	Central Neuron of NPY/AgRP, POMC Anterior pituitary Effects: ↑appetite, pituitary growth hormone ↓energy consumption	Central kisspeptin in H, anterior pituitary Effects Regulating the release of GnRH, FSH and LH	Andrews (2011), Fernández-Fernández et al. (2005), Frazao et al. (2014), López and Nogueiras (2023), Pan et al. (2020)
			Peripheral pancreatic β cells, hepatocytes, adipocytes, etc. Effects ↓insulin synthesis and secretion ↑white adipose tissue synthesis	Peripheral oocytes, luteum and stromal cells Effects ↓development of follicles and ovulation ↓progesterone production	López and Nogueiras (2023), Tropea et al. (2007)
GLP-1	Small Intestinal L cell	Camp-PKA-Creb	Central H, hindbrain and other brain areas Effects ↓appetite and eating ↑adipose decomposition	Central Neuron of kisspeptin, GnRH, P Effects Regulating the secretion of GnRH, LH and FSH	Farkas et al. (2016), Outeiriño-Iglesias et al. (2015), Arbabi et al. (2021), Bu et al. (2024), Ibrahim et al. (2024)
			Peripheral: pancreatic islets β cells gastrointestinal intramuscular plexus, etc. Effect ↑insulin secretion, ↓BG, gastrointestinal peristalsis, gastrointestinal digestion and absorption function, ↑satiety and so on	Peripheral: ovarian, granulosa cells Effect Influence the secretion of progesterone and the luteinization of LH in granulosa cells	Khan et al. (2022), Nishiyama et al. (2018), Ussher and Drucker (2023)
Growth hormone	Anterior Pituitary	JAK-STAT	Peripheral: adipose cells, liver, muscle, pancreas, etc. Effects ↑glycogen liver glycogenolysis and gluconeogenesis, lipolysis in adipocytes, insulin secretion, ↓glucose uptake in muscle, induce systemic IR	Central: Neuron of kisspeptin, GnRH. Effect	Bhattarai et al. (2010), Martínez-Moreno et al. (2018)
				Synergistic with IGF- 1 ↑GnRH release Peripheral: ovarian oocytes, granulosa cells, vascular endothelial cells, etc. Effects ↑the FSH expression, estrogen synthesis of granulosa cells, luteinization of granulosa cells and maintain luteal function ↑ovarian angiogenesis	Devesa and Caicedo (2019), Kopchick et al. (2020), Tidblad (2022)

↑ means promote, ↓ means reduce and inhibit, H: hypothalamus, P: pituitary, GnRH: gonadotropin-releasing hormone, BG: blood glucose, IR: insulin resistance.

## 3 Obesity-related reproductive endocrine disorders in women

From the perspective of clinical research evidence, the risk of anovulatory infertility increases proportionally with higher BMI, highlighting the significant influence of BW on reproductive health (Rich-Edwards et al., 1994). Specifically, when BMI exceeds 29, the probability of conception decreases by approximately 5% (van der Steeg et al., 2008), while each additional unit increase in BMI reduces the likelihood of achieving pregnancy via *In Vitro* Fertilization (IVF) by 2.2%–4.3%. Consequently, approximately 33% of obese women fail to conceive naturally even after 1 year of attempting (Lake et al., 1997). Obesity also leads to complications in pregnancy outcomes (Practice Committee of the American Society for Reproductive MedicinePractice Committee of the American Society for Reproductive Medicine, 2021). PCOS is responsible for 80% of anovulatory infertility cases in women, with hyperandrogenism (HA) observed in 60%–80% of affected individuals (Azziz et al., 2006; Balen et al., 2016). The condition is notably more prevalent among obese women, who are 30% more likely to develop PCOS compared to non-obese women. In fact, up to 60% of women with PCOS are affected by overweight or obesity, highlighting the strong association between BW and this disorder (Teede et al., 2021). After accounting for the influence of childhood weight, the risk of menstrual irregularities in obese women increases by 1.97 times, with the prevalence of amenorrhea or oligomenorrhea also increasing alongside higher BMI. Studies have shown that obesity at age 7 is an independent predictor of menstrual problems later in life, such as at age 33 (Lake et al., 1997). Notably, central obesity is a better predictor of ovulatory dysfunction than overall BF, with women experiencing anovulation typically presenting with larger Waist circumference (WC) compared to ovulating women with similar BMI (Zaadstra et al., 1993). In addition, central precocious puberty (CPP), caused by the early activation of the HPO axis, is strongly associated with increased BMI. Overweight or obese children are nearly twice as likely to develop CPP compared to their normal-weight peers (Biro et al., 2010; Liu et al., 2021).

Obesity has been identified as an independent risk factor for premature ovarian insufficiency (POI), characterized by a significant decline in ovarian function before the age of 40. This is supported by a multicenter cross-sectional study involving participants from eight European countries, which also found that maintaining a normal weight and never smoking are protective factors (Vogt et al., 2022). While the exact mechanisms by which obesity contributes to POI remain unclear, obesity and POI likely share common risk factors. For instance, excessive exposure to environmental endocrine disruptors is implicated in both the development of obesity and the onset of POI and early menopause (Ding et al., 2022). Additionally, a higher BMI is associated with an earlier age at menarche, which in turn is a recognized risk factor for early menopause (Juul et al., 2017; Zhang X. et al., 2023). Obesity also increases the risk and severity of endometriosis, further linking it to early menopause and POI (Trabert et al., 2011; Venkatesh et al., 2022).

## 4 Mechanism of obesity on female reproductive endocrine

### 4.1 Mechanisms in the brain level

#### 4.1.1 Female HPO axis and the obesity influence on the production of pituitary gonadotrophin

**FIGURE 1:**
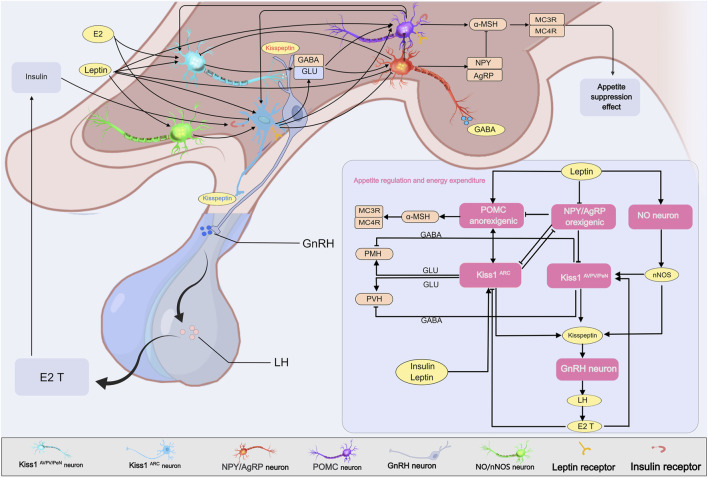
Dual regulatory mechanism on reproduction and metabolism. The hypothalamic kisspeptin neurons are expressed in ARC and AVPV/PeN regions, releasing kisspeptin to influence GnRH neurons. Kisspeptin neurons project to POMC and NPY/AgRP neurons and co-express glutamate in the ARC region and GABA in the AVPV/PeN region, thus regulating appetite and metabolism. POMC and NPY/AgRP neurons also interact with kisspeptin neurons, establishing a neural mechanism that integrates metabolic processes with reproductive function. ARC: arcuate nucleus, AVPV/PeN: anteroventral periventricular nucleus/periventricular nucleus, GnRH: gonadotropin-releasing hormone, POMC: pro-opiomelanocortin, NPY/AgRP: neuropeptide Y/agouti-related peptide, PVH: paraventricular hypothalamic nucleus, DMH: dorsomedial hypothalamus, nNOS: neuronal nitric oxide synthase, NO: nitric oxide, LH: luteinizing hormone, E2: estradiol, T: testosterone.

Obesity affects reproductive health by disrupting the HPO axis. Kiss1 neurons located in the hypothalamus, responsible for producing kisspeptin, are crucial in regulating the secretion of gonadotropin-releasing hormone (GnRH) and the release of gonadotropins. Kiss1 neurons function as metabolic sensors, linking energy balance to reproductive functions (Navarro, 2020). There are two distinct populations of kiss1 neurons, including those located in ARC (Kiss1^ARC^) and those in anteroventral periventricular/periventricular nucleus (Kiss1^AVPV/PeN^) of the hypothalamus. Ovarian steroids differentially regulate them. For instance, estradiol (E2) upregulates the production of kisspeptin in Kiss1^AVPV/PeN^ neurons while downregulating it in Kiss1^ARC^ neurons (Uenoyama et al., 2021). Additionally, Kiss1^ARC^ neurons co-express glutamate, while Kiss1^AVPV/PeN^ neurons co-express gamma-aminobutyric acid (GABA), with both neurotransmitters being upregulated by E2 in females. Furthermore, Kiss1^ARC^ neurons co-express receptors for leptin and insulin, and are activated by these hormones in a state of satiety. Kiss1^ARC^ neurons also stimulate anorexigenic POMC neurons while inhibiting orexigenic neuropeptide Y (NPY)/agouti-related peptide (AgRP) neurons, linking feeding behavior to reproductive functions (Navarro, 2020). Both Kiss1^ARC^ and Kiss1^AVPV/PeN^ neurons project to the paraventricular hypothalamic nucleus (PVH) neurons involved in satiety and the dorsomedial hypothalamus (DMH) neurons responsible for regulating energy expenditure, modulating their functions through the release of glutamate and GABA, with an upregulation by E2 in females (Figure 1; Rønnekleiv et al., 2022).

As a consequence of Kiss1 neuron dysfunction, obesity significantly affects GnRH-LH release patterns in females. Compared to women of normal weight, obese women exhibit significantly lower average LH levels when measured every 10 min over 12 h, with an exceedance of a 50% reduction in LH pulse amplitude during the follicular phase (Jain et al., 2007). This reduction results in inadequate corpus luteum formation and lower progesterone (P) production by the ovaries. Although obese women with lower follicular phase LH levels have similar serum E2 levels compared to non-obese women, obese women during ovulation exhibit a reduced LH surge, resulting in inadequate corpus luteum formation and lower mid-luteal P production (luteal phase deficiency) (Jain et al., 2007). While increased GnRH/LH pulse frequency is observed in obese individuals and those with PCOS, obesity primarily affects LH pulse amplitude rather than frequency, leading to an overall decrease in mean LH levels (Morales et al., 1996). In women with oligomenorrhea and anovulatory PCOS, both mean LH levels and LH pulse amplitude negatively correlate with BMI, further suggesting that obesity-induced metabolic changes contribute to reproductive endocrine dysfunction (Taylor et al., 1997).

#### 4.1.2 Brain insulin resistance

In women with obesity, metabolic imbalances such as IR and increased adiposity alter the function of these hypothalamic neurons, leading to disrupted reproductive signaling. IR can impair hypothalamic insulin responsiveness, a phenomenon known as “brain IR” (Milstein and Ferris, 2021). Exposure to an HFD impairs ARC neurons, contributing to neuronal fibrosis, reduced insulin receptor activation in these hypothalamic neurons, and worsening brain IR (Beddows et al., 2024). Disruption of insulin signaling in neurons leads to reproductive dysfunction, such as reduced LH levels and infertility, as demonstrated by studies where InsR (insulin receptor) knockout in neural stem cells during early brain development is mediated by Nestin-Cre (Brüning et al., 2000). However, in models with widespread deletion of InsR in the brain, the pituitary’s responsiveness remains normal, indicating that the impact on LH levels may result from dysregulation of hypothalamic GnRH production rather than a direct pituitary defect (Brüning et al., 2000), thereby supporting the hypothesis that the hypothalamus is a key brain region for insulin’s action (Milstein and Ferris, 2021).

Insulin has been shown to affect reproductive function by directly modulating LH secretion. For example, injection of insulin into the lateral ventricle of insulin-deficient diabetic sheep and rats increases both the frequency and peak levels of LH pulses (Kovacs et al., 2002; Tanaka et al., 2000). However, insulin’s effects on GnRH production may be indirect, as conditional knockout of InsR in GnRH neurons does not affect puberty onset, estrous cycles, or litter size in female mice (Divall et al., 2010). This suggests that insulin may regulate reproductive function through other neural circuits. One such circuit involves kiss1 neurons, which are crucial for initiating the secretion of GnRH. Although only a subset of kiss1 neurons in female mice express InsR—approximately 22% in the ARC and 3%–5% in the periventricular region—insulin signaling in these neurons is critical for reproductive health (Qiu et al., 2013). Specific knockout of InsR in kiss1 neurons results in reduced LH levels and delayed puberty onset in female mice, although fertility and estrous cycles remain unaffected (Qiu et al., 2013). Interestingly, when IGF-1 receptors are also knocked out in kiss1 neurons, a reduction in litter size is observed, suggesting that insulin and IGF-1 signaling cooperate in these neurons to regulate reproductive function (Wang M. et al., 2024).

Interestingly, the crosstalk between IR and steroid hormone imbalance is also a key mechanism causing HPO axis dysfunction. In obese individuals, elevated insulin levels promote androgen production, which is then converted into additional estrogen by aromatase in adipocytes (Cirillo et al., 2008). Excess estrogen reduces LH production through negative feedback on the HPO axis, further impairing reproductive function. A study supporting this finding shows that aromatase inhibitors significantly increase LH pulse amplitude by 2.54-fold in obese women, a response not observed in women of normal weight (Venkatesh et al., 2022).

#### 4.1.3 Brain leptin resistance

Leptin resistance (LR) is another characteristic feature of obesity, accompanied by elevated serum leptin levels (Izquierdo et al., 2019). The hypothalamus serves as the central target for leptin in regulating feeding, energy metabolism and reproductive functions. Although leptin overexpression accelerates puberty onset and enhances reproductive capacity in young female mice, prolonged hyperleptinemia eventually leads to hypothalamic hypogonadism characterized by prolonged estrous cycles, ovarian atrophy and impaired GnRH and LH secretion (Yura et al., 2000).

Leptin primarily exerts its effects through two types of neurons in the ARC of the hypothalamus: POMC and NPY/AgRP neurons. These neurons have opposing roles in regulating appetite and energy metabolism. POMC neurons release α-melanocyte-stimulating hormone (α-MSH), which acts on melanocortin receptors (MC3R and MC4R) in the hypothalamic preoptic area to produce anorexigenic effects. In contrast, NPY/AgRP neurons inhibit POMC activity by secreting AgRP, NPY, and GABA, counteracting the anorexigenic effect of POMC (Cowley et al., 2001; Schwartz et al., 1996; Stephens et al., 1995). Both POMC and NPY/AgRP neurons are interconnected with kisspeptin neurons, establishing a neurobiological link between metabolic regulation and reproduction. NPY/AgRP neurons inhibit kisspeptin neurons in the ARC and AVPV/PeN region of the hypothalamus, thereby reducing kisspeptin production and subsequently decreasing GnRH and LH secretion (Coutinho et al., 2020; Padilla et al., 2017). Conversely, POMC neurons activate Kiss1^ARC^ neurons, thereby promoting the release of GnRH and LH (Israel et al., 2012). Notably, kisspeptin and GnRH neurons either do not express LepR or express them at very low levels, suggesting that leptin likely influences the HPO axis predominantly through its effects on POMC and NPY/AgRP neurons (Louis et al., 2011; Figure 1). Experimental studies have shown that knockout of LepR specifically in AgRP neurons results in reduced LH production, arrested estrous cycles, and decreased fertility in female mice (Egan et al., 2017). These studies highlight the crucial role of NPY/AgRP neurons in mediating leptin’s impact on reproduction. Furthermore, hypothalamic nitric oxide (NO) neurons, which also express LepR, appear to play a role in leptin’s central regulation of the HPO axis. Approximately 20% of LepR-expressing neurons in the hypothalamus also produce NO via neuronal nitric oxide synthase (nNOS). Knockout of LepR in these NO neurons results in hyperphagic obesity, reduced energy expenditure, and hyperglycemia similar to what is observed in global LepR-deficient mice (Leshan et al., 2012).

On the other hand, NO, as a lipophilic gaseous molecule that senses leptin signals in the brain, can freely diffuse and act on neighboring neurons (such as GnRH neurons), regulating their pulsatile secretion activity. By knocking out the nNOS gene (nNOS^−/−^) or pharmacologically inhibiting the activity of nNOS in the hypothalamic preoptic area, the promoting effect of exogenous leptin on LH secretion is significantly weakened, and leptin cannot restore the fertility of leptin-deficient female mice, proving that the nNOS/NO pathway is a necessary condition for leptin to regulate reproduction (Bellefontaine et al., 2014). Mechanistically, NO may directly activate the soluble guanylate cyclase (sGC)-cGMP pathway in GnRH neurons, modulating their excitability, or regulate the preoptic local neural circuitry (including Kisspeptin neurons or GABA/glutamatergic interneurons), indirectly controlling the GnRH pulse generator (Bellefontaine et al., 2014).

In summary, LR and excess leptin disrupt the functions of intermediate neurons, such as POMC, NPY/AgRP, and nNOS neurons. Through pathways including leptin-melanocortin-kisspeptin, leptin-NPY/AgRP-kisspeptin and leptin-nNOS/NO-kisspeptin, leptin influences both energy metabolism and GnRH release by modulating hypothalamic kisspeptin-producing neurons (Padilla et al., 2017; Leshan et al., 2012; Constantin et al., 2021; Hessler et al., 2020; Hill et al., 2010). Kisspeptin is thus considered a crucial link between leptin signaling and GnRH secretion, as well as a key integrator of metabolic energy homeostasis and reproductive function (Figure 1).

#### 4.1.4 Hypothalamic inflammation

In individuals with obesity, inflammation is primarily driven by increased levels of inflammatory markers and activated signaling pathways, which contribute to systemic organ dysfunction (Cox et al., 2015). Obesity and prolonged HFD exposure result in chronic systemic inflammation, which significantly disrupts hypothalamic function and impairs reproductive endocrine function, indicating the hypothalamus is particularly vulnerable to obesity-induced inflammation (Sewaybricker et al., 2023). In the context of obesity, macrophages play a crucial role in driving systemic inflammation. These immune cells are abundant in adipose tissue, particularly visceral fat, and release inflammatory cytokines and other mediators that promote both peripheral and central inflammation (Weisberg et al., 2003). Although the blood-brain barrier (BBB) traditionally protects the brain from direct infiltration by peripheral immune cells, evidence suggests that macrophages from adipose tissue can infiltrate into the hypothalamus during obesity, thereby contributing to local inflammation (Valdearcos et al., 2017).

Despite the involvement of peripheral macrophages, resident central nervous system (CNS) immune cells, particularly microglia, appear to have a more prominent role in maintaining chronic hypothalamic inflammation. In obesity, hypothalamic microglia are activated by elevated circulating saturated fatty acids via the Toll-like receptor 4 (TLR-4)/NF-κB signaling pathway, leading to the polarization of microglia into a pro-inflammatory M1 macrophage-like phenotype, which exacerbates local inflammation in the hypothalamus (Lively and Schlichter, 2018). Recent studies have shown that depleting or inhibiting microglial activation can reduce BW, food intake, and peripheral macrophage infiltration, highlighting the close interaction between peripheral and central immune cells in sustaining hypothalamic inflammation (Valdearcos et al., 2017).

**FIGURE 2:**
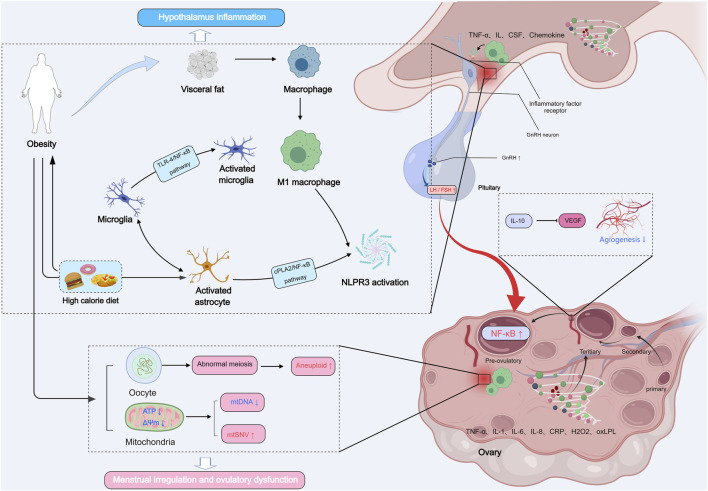
Obesity induces dysfunction in GnRH neurons mediated by hypothalamic inflammatory pathways. Macrophages in visceral adipose tissue are a key source of inflammatory factors, influencing microglia and astrocytes and releasing inflammatory factors, thereby impacting GnRH neurons and the ovarian microenvironment (oocyte meiosis, mitochondrial function, and angiogenesis).

Astrocytes, another type of glial cell, also play a crucial role in hypothalamic inflammation. Under conditions such as autoimmune encephalopathy, brain injury, or microbial infections, astrocytes are activated and produce a range of inflammatory factors via the cytosolic phospholipase A2 (cPLA2)-NF-κB signaling pathway. Activated astrocytes release various inflammatory factors, including TNF, ILs, chemokine ligands, and colony-stimulating factors, which interact with microglia, oligodendrocytes, and neurons, influencing brain pathology and recovery (Linnerbauer et al., 2020). Disruption of NF-κB signaling in astrocytes has been shown to ameliorate HFD-induced hypothalamic inflammation, reduce weight gain, and improve glucose tolerance, highlighting the importance of astrocytic regulation in hypothalamic responses to obesity (Figure 2; Douglass et al., 2017).

Hypothalamic inflammation caused by obesity and HFD has profound consequences for reproductive health, which is commonly associated with menstrual irregularities and ovulatory dysfunction, such as those observed in PCOS (Barlampa et al., 2021). GnRH neurons express receptors for various inflammatory mediators, including interleukins, prostaglandins (PGEs), and TNF-α, suggesting that these neurons are directly regulated by inflammatory factors (Jasoni et al., 2005). For instance, IL-10 knockout results in impaired GnRH secretion and loss of estrous cycles (Barabás et al., 2018). In addition to these direct effects, inflammatory factors such as TNF-α and bacterial endotoxins, like lipopolysaccharide (LPS), can also indirectly influence kisspeptin neurons in the hypothalamus, thereby impairing GnRH secretion and further contributing to reproductive dysfunction (Lee et al., 2019; Sarchielli et al., 2017). The interplay between inflammation, kisspeptin neurons, and GnRH release highlights that inflammation can disrupt both the neural networks regulating reproductive hormone release and the feedback mechanisms necessary for normal reproductive function.

### 4.2 Mechanisms in the ovary level

#### 4.2.1 Oocyte meiosis

The ovaries are the primary organs responsible for producing oocytes and hormones, such as E2 and P, which are crucial to reproductive health. Ovarian function directly determines reproductive potential. Although the ovaries can still function to some extent in the absence of hypothalamic and pituitary function, obesity disrupts ovarian processes, directly impairing follicle development and oocyte quality. Oocyte meiosis is a critical process for oocyte maturation and successful fertilization. Oocytes are temporarily arrested at metaphase I of meiosis, during which the nuclear envelope remains intact, and are referred to as germinal vesicle (GV) stage oocytes. The resumption of meiosis involves the breakdown of the germinal vesicle (GVBD) and the first meiotic division, leading to spindle reorganization and ultimately, fertilization (Pan and Li, 2019). Spindle formation is critical for the completion of meiosis and oocyte maturation. In HFD-induced obese mice, oocytes exhibit reduced GVBD rates, abnormal spindle morphology, chromosome misalignment, and disrupted oocyte polarization, which contribute to increased rates of aneuploidy (Figure 2; Hou et al., 2016). Similarly, obese women undergoing IVF exhibit a higher incidence of spindle abnormalities and chromosomal misalignments in oocytes, leading to impaired oocyte maturation (Gonzalez et al., 2022; Machtinger et al., 2012). Furthermore, studies have shown that obesity alters gene expression in oocytes, upregulating CXCL2 and DUSP1, while downregulating TWIST1, ID3, GAS7, and TXNIP. These genes are involved in inflammation, oxidative stress, and lipid metabolism (Ruebel et al., 2017). These findings suggest that obesity impairs oocyte maturation and quality through both genetic and epigenetic alterations, including changes in DNA methylation and histone acetylation, which negatively affect chromatin stability during meiosis (Hou et al., 2016; Yun et al., 2019).

#### 4.2.2 Mitochondrial damage in oocytes

Mitochondria are crucial for oocyte quality, as they provide the energy needed for oocyte maturation, fertilization, and embryonic development (Bahety et al., 2024). Oocyte quality is closely associated with mitochondrial DNA (mtDNA) copy number and mitochondrial function. Primary oocytes exhibit substantial mtDNA expansion during early maturation, and oocytes with higher mtDNA copy numbers have a greater likelihood of fertilization success (Cao et al., 2007; Reynier et al., 2001). Additionally, Mitochondrial membrane potential and ATP production are also critical for oocyte quality (Figure 2; Acton et al., 2004). In obese female mice, oocytes exhibit mitochondrial dysfunction, including reduced mtDNA copy number, increased mtDNA mutations, impaired mitochondrial membrane potential, and elevated autophagy levels (Wu et al., 2015). Furthermore, oocytes from obese mice have diminished mitochondrial density, inhibited mitochondrial membrane potential, and accumulation of abnormal mitochondrial aggregates (Chen et al., 2024). Moreover, mature oocytes from obese mice show reduced mtDNA and increased mitochondrial single-nucleotide variant (mtSNV) rates, impairing mitochondrial energy function and oocyte quality (Chen et al., 2024). Obesity-induced damage to oocyte mitochondria may be related to the inhibition of AMPK activity, leading to increased binding affinity of the ATF5-POLG protein complex to the mutated mtDNA D-loop and protein-coding regions, thereby causing the replication of heteroplasmic mtDNA (Chen et al., 2024). Interestingly, mitochondrial damage in oocytes from obese mothers may also affect offspring, passing down metabolic and mitochondrial dysfunction across generations (Elías-López et al., 2023).

#### 4.2.3 Granulosa cells

Granulosa cells, essential components of follicles, produce estrogen and play a vital role in oocyte differentiation and ovulation. In both individuals with obesity and PCOS, granulosa cells exhibit reduced proliferation, increased apoptosis, and impaired steroidogenesis, negatively affecting follicle maturation and oocyte quality (Nteeba et al., 2014; Peng et al., 2021). These abnormalities are often accompanied by elevated oxidative stress, mitochondrial dysfunction, decreased ATP levels, endoplasmic reticulum stress, and autophagy dysfunction in granulosa cells (Zhao et al., 2023). Obesity-induced lipotoxicity and metabolic dysfunction in granulosa cells contribute to these impairments, reducing female fertility (Hua et al., 2020). Additionally, certain non-coding RNAs, such as miR-133a, play a role in obesity-induced granulosa cell apoptosis, targeting typical anti-apoptotic genes, including C1QL1 and XIAP, and pro-apoptotic genes, such as PTEN (Chen et al., 2023). Studies have also revealed that obesity affects granulosa cells at various stages of follicle development. For example, in obese mice, excessive proliferation of granulosa cells in primordial follicles accelerates follicular depletion, mimicking PCOS-like ovarian phenotypes and reducing ovarian reserve (Zhou et al., 2023). This study employed laser capture microdissection and RNA sequencing to dissect and analyze primordial and primary follicles at various developmental stages, aiming to identify gene expression changes during the transition from primordial to primary follicles (PFT). The results showed significant increases in ferroptosis, oxidative stress, vascular endothelial growth factor, and mTOR signaling markers in primordial follicles from obese mice, suggesting that increased lipid metabolism-related ferroptosis in obesity may be a key mechanism for excessive activation of primordial follicles (Zhou et al., 2023). Recent single-cell sequencing (scRNA-seq) studies have found that in both diet-induced and leptin-deficient obese mice, the granulosa cell subtype expressing inhibin B increases, and pseudo-temporal analysis has shown that this granulosa cell subtype is mainly distributed in more mature antral follicles. Moreover, obesity induces a shift in granulosa cell subtypes, contributing to follicular arrest and impaired follicle maturation, which is further influenced by altered androgen production by theca cells in obese ovaries (Long et al., 2022; Salilew-Wondim et al., 2015).

#### 4.2.4 Ovarian microenvironment

**FIGURE 3:**
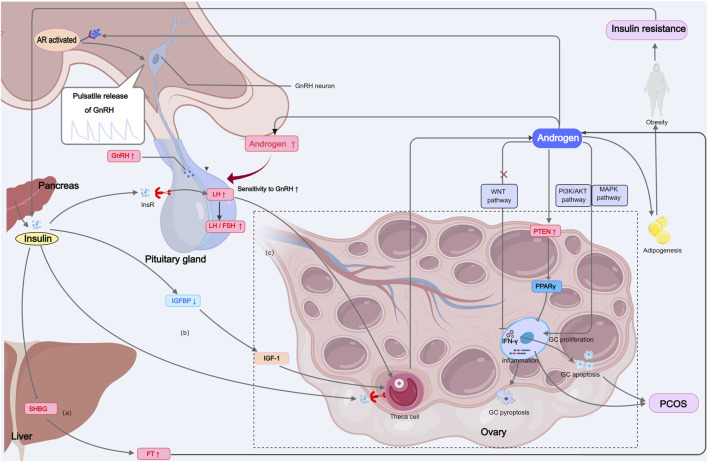
IR and HA constitute the pathophysiological basis of metabolic and reproductive disorders in PCOS. IR and hyperinsulinemia increase free testosterone by inhibiting SHBG production, acting on InsR in theca cells or enhancing IGF-1 stimulation, which increases androgen secretion. Additionally, central actions increase LH secretion. Excessive androgens activate PI3K/AKT, MAPK, PTEN, and downregulate WNT signaling pathways, leading to increased GC proliferation, reduced differentiation, follicular arrest, and polycystic ovary formation. Androgens can also suppress IFN-γ expression and promote apoptosis in GCs. At the same time, excess androgens can exacerbate abdominal obesity, IR, and systemic inflammation. SHBG: sex hormone-binding globulin, IR: insulin resistance, InsR: Insulin Receptor, IGF-1: insulin-like growth factor 1, LH: Luteinizing Hormone, PCOS: polycystic ovary syndrome, AR: androgen receptor, IGFBP: insulin-like growth factor-binding proteins, GC: granulosa cell.

The ovarian microenvironment (OME), comprising follicular fluid, stroma, vasculature, and immune cells, plays a crucial role in regulating ovarian functions, including follicular development, hormone production, and oocyte maturation (Duffy et al., 2019; Shen et al., 2023). Chronic low-grade inflammation in the ovarian microenvironment is a key factor contributing to follicular dysfunction in obesity. Elevated levels of pro-inflammatory cytokines (e.g., TNFα, IL-6, IL-8) and oxidative stress markers in the ovaries of obese individuals disrupt normal ovarian function and accelerate ovarian aging (Snider and Wood, 2019; Xiong et al., 2011). The excessive pro-inflammatory factors (IL-1, IL-6, TNFα, and CRP) and oxidative stress factors (H2O2, oxLDL) in the ovarian tissue and follicular fluid of obese individuals may be produced by follicular cells and immune cells within the ovary (Ruebel et al., 2017; Nteeba et al., 2014) or be associated with elevated circulating inflammatory factors in obese individuals (Figure 3). Therefore, systemic inflammation in obese individuals can be transmitted to the ovaries and even the follicular fluid, thereby affecting ovarian function.

Obesity also leads to dysregulation of ovarian angiogenesis, which is essential for supplying nutrients and oxygen to developing follicles. During early development, small follicles lack their own vascular network and rely on stromal vessels for nutrients and oxygen. As follicles mature, each creates its own vascular network within the theca layer, ensuring an independent supply of nutrients and oxygen separate from other follicles. Alterations in angiogenesis can lead to the formation of abnormal vascular structures, which can negatively impact follicle development and ovulation (Duncan and Nio-Kobayashi, 2013). In addition to chronic inflammation, dysregulation of angiogenesis has also been observed in the ovaries of obese and PCOS individuals (Di Pietro et al., 2018). For example, increased neovascularization and an imbalance of pro/antiangiogenic factors are present in the ovarian stroma of PCOS, and this dysregulation of angiogenesis is thought to contribute to the characteristic ovarian features of PCOS, such as abnormal follicular development, increased numbers of small follicles, failure of dominant follicle selection, anovulation, and the formation of follicular cysts (Di Pietro et al., 2018). Angiogenic factors, such as vascular endothelial growth factor (VEGF), not only stimulate vascular formation but also directly affect theca cells or granulosa cells, influencing follicular development and their endocrine function (Irusta et al., 2010). In high-fat and fructose diet-induced obese mice, early ovarian follicle accumulation and reduced numbers of mature follicles and corpora lutea are accompanied by a decrease in the number of microvessels in early follicles. This phenotype may be related to the excessive expression of IL-10 in the periovarian adipose tissue during obesity, which disrupts the function of VEGF and contributes to ovarian aging. This effect can be mitigated by treatments such as metformin (Yang et al., 2021). Recent single-cell and spatial transcriptomic sequencing studies have provided high-resolution cellular maps of the impact of obesity on the ovarian microenvironment. The study found that genetic obesity of OB/OB mice, but not HFD-induced obesity, significantly altered the proportions of granulosa cells, theca-stroma cells, luteal cells, and vascular endothelial cells in the ovary. Obesity severely disrupted granulosa cell differentiation from small to large follicles. Functionally, HFD enhanced FSH sensitivity and related hormone production, whereas OB/OB mice had decreased FSH sensitivity, insufficient steroid hormone production, and impaired follicular development. These differences can be attributed to the distinct expression patterns of the transcription factor Foxo1 in the two types of obese mice (Jiang et al., 2024).

#### 4.2.5 Obesity and ovarian aging

In animal experiments, the effects of obesity on ovarian reserve function have shown variability. In diet-induced obesity (e.g., high-fat and high-carbohydrate diets) in rodents, rapid depletion of ovarian reserves has been observed (Wang et al., 2014). Similar findings have been reported in rabbit studies (Díaz-Hernández et al., 2022). Conversely, caloric restriction or maintaining optimal nutrition can reduce primordial follicle activation, increase the number of quiescent primordial follicles, prolong reproductive lifespan, and delay the onset of menopause (Garcia et al., 2019). Compared to caloric-restricted rats, diet-induced obese rats exhibit mTOR-related signaling associated with aging, along with decreased expression of anti-aging molecules such as SIRT1, SIRT6, FOXO3a, and NRF-1 (Wang et al., 2014). Enhanced mTOR signaling is also associated with the overactivation and depletion of primordial follicles, suggesting that obesity may accelerate ovarian reserve depletion by excessively activating mTOR pathways (Guo and Yu, 2019). These findings align with the conclusions drawn from studies using laser capture microdissection and RNA sequencing to investigate the characteristics of the primordial to primary follicle transition in obese mice, which suggest that obesity may accelerate primordial follicle pool depletion and decline in ovarian reserve function through excessive activation of mTOR signaling (Zhou et al., 2023). In OB/OB mice, despite increased follicular atresia, reduced numbers of pre-ovulatory follicles (large antral follicles), and a significant decrease in the number of corpora lutea, the expression of markers related to ovarian reserve, including Dazl, Stra8, and ZP3 mRNA, are increased. Additionally, the count of primordial follicles is also elevated, suggesting that leptin deficiency may have a protective effect on ovarian reserve under certain genetic conditions (Mollah et al., 2021). The differences in the effects between diet-induced and genetic obesity on ovarian reserve may be influenced by leptin, as diet-induced obesity is often accompanied by elevated circulating leptin levels, which may accelerate follicular overactivation through stimulation of the HPO axis. Elevated leptin might also directly affect granulosa cells by inhibiting anti-Müllerian hormone (AMH) expression, contributing to excessive follicle depletion (Merhi et al., 2013).

In addition to reduced ovarian reserve, increased extracellular matrix deposition, fibrosis, and the accumulation of senescent cells are also hallmarks of ovarian aging. These changes are also commonly observed in PCOS (Zhou et al., 2017). Chronic low-grade inflammation in the ovary is believed to contribute to these ovarian aging phenotypes (Isola et al., 2024). Increased ovarian fibrosis and the accumulation of senescent cells have been observed in HFD-induced obese rats and OB/OB mice, characterized by increased expression of p21 and p16, increased lipofuscin staining, and macrophage infiltration (Kawai et al., 2021). Additionally, ovarian fibrosis phenotypes similar to those observed in age-dependent ovarian aging have been reported in genetic obesity mice with Alms1 gene mutations (Umehara et al., 2022). Macrophages may play an important role in mediating inflammation-induced ovarian fibrosis and aging. The pro-inflammatory M1 macrophage phenotype is predominant in obese and PCOS ovaries (Feng et al., 2023) and promotes granulosa cell apoptosis and follicular atresia through the production of cytokines such as TNF-α, IL-1α/β, IL-6, and IL-18, thereby promoting the high expression of inflammasome genes such as NLRP3 and apoptosis associated speck like protein containing caspase activation and recruitment domain (ASC). During the later stages of reproductive age, increased extracellular matrix and fibrosis may also be linked to the M2 macrophage subtype, which produces factors such as TGF-β, FGF, and PDGF, as well as pro-inflammatory cytokines like IL-6, that contribute to fibrosis (Vasse et al., 2021).

Besides, both obesity and aging can induce similar pathological changes in the ovarian microenvironment, such as mitochondrial dysfunction, endoplasmic reticulum stress, oxidative stress, liptoxicity and inflammation (Wu et al., 2015; Snider and Wood, 2019; Tatone et al., 2008). The anti-fibrotic drug BGP-15 can reverse obesity- and aging-induced ovarian fibrosis by inhibiting M2 macrophage polarization and MMP13 protein upregulation, as well as correcting mitochondrial dysfunction, oxidative damage, and ER stress in the ovarian stroma (Umehara et al., 2022). Interestingly, metformin, a typical anti-inflammatory and anti-aging drug, has also been found to prevent age-dependent ovarian aging in mice by altering the functional subpopulations of macrophages and fibroblasts in the ovary, and it can also reverse premature ovarian fibrosis in obese mice (Umehara et al., 2022; Landry et al., 2022).

### 4.3 The systematic mechanism by which obesity affects female reproductive endocrine from the perspective of PCOS

#### 4.3.1 The crosstalk between obesity and HA

PCOS is a common reproductive endocrine-metabolic disease among adolescent and reproductive-age women, which is characterized by infrequent ovulation, menstrual disorders, and HA, and is commonly associated with obesity, IR, and HA. The coexistence of these phenotypes in PCOS can be understood as an adaptive response to adverse environments such as resource scarcity. Elevated androgen levels, in particular, may represent an evolutionary adaptation that enabled females to engage in survival activities, such as hunting or defending against predators, by enhancing energy mobilization and physical resilience (Parker et al., 2022). Additionally, IR can also be present in individuals with PCOS who have a normal BW (Diamanti-Kandarakis and Dunaif, 2012). In women with PCOS, IR and hyperinsulinemia are key contributors to elevated androgen levels through multiple mechanisms: a) Insulin inhibits hepatic production of sex hormone-binding globulin (SHBG), leading to increased circulating free testosterone levels (Preziosi et al., 1993); b) Insulin acts directly on the InsR in theca cells or enhance the effect of IGF-1 on theca cells by reducing insulin-like growth factor-binding proteins (IGFBPs), thereby increasing LH-dependent ovarian androgen production (Franks and Hardy, 2018); c) Insulin can centrally stimulate the pituitary to secrete more LH, further promoting excessive androgen production by theca cells and ovarian stromal cells (Figure 3).

In a physiological context, androgens play a crucial role in female reproductive health, bone integrity, and cognitive function (Bianchi et al., 2021). However, excessive androgen levels in women can result in systemic damage. HA promotes the differentiation of preadipocytes into mature adipocytes, resulting in adipocyte hypertrophy and central obesity. This exacerbates dyslipidemia, oxidative stress, and systemic inflammation, which in turn worsen both obesity and IR (Lonardo et al., 2024). The systemic effects of elevated androgens are linked to an increased risk of CVD, NAFLD, T2DM, and malignancies in PCOS patients (Ye et al., 2021). Moreover, excessive androgen exposure *in utero* can predispose female offspring to obesity, PCOS, and other metabolic disorders in adulthood (Abbott et al., 1998; Nohara et al., 2013; Padmanabhan et al., 2010; Recabarren et al., 2005). Additionally, elevated androgens also disrupt the HPO axis, impairing ovulation and reproductive function. Although androgen receptor (AR) expression is higher in the male brain compared to the female brain, ARs are also present in the hypothalamus and extrahypothalamic nuclei of females, including fetal female mice (Handa et al., 1986). Under hyperandrogenic conditions, AR activation in the hypothalamus may induce inflammation (Ubba et al., 2023) and alter the firing frequency of GnRH neurons, thereby affecting their pulsatile release through GABA signaling, which likely increases LH pulse frequency not via kisspeptin pathways (Moore et al., 2015). At the pituitary level, AR activation enhances the pituitary’s sensitivity to GnRH, particularly increasing LH secretion, leading to the characteristic elevated LH/FSH ratio seen in PCOS (Schanbacher et al., 1987). At the ovarian level, excess androgens suppress granulosa cell proliferation through mechanisms such as upregulating phosphatase and tensin homolog (PTEN) expression, which is PPARγ-dependent, or by disrupting WNT signaling, both of which lead to follicular arrest and polycystic ovary formation (Chen et al., 2015; McFee et al., 2021). Additionally, androgens regulate granulosa cell apoptosis through inflammatory pathways, promoting inflammasome expression and pyroptosis (Wang et al., 2020). Androgens can also inhibit granulosa cell proliferation by suppressing interferon-gamma (IFN-γ) expression, thereby contributing to ovarian dysfunction in PCOS (Li Y. et al., 2019; Figure 3). Interestingly, studies suggest that neuron-specific AR signaling may play a more critical role in the development of PCOS phenotypes than peripheral AR (Caldwell et al., 2017). For instance, mice with neuron-specific AR deletion (NeuARKO) are resistant to developing the PCOS phenotype induced by dihydrotestosterone (DHT) (Caldwell et al., 2017). In contrast, global AR knockout mice (ARKO) treated with testosterone exhibit normal estrous cycles and corpus luteum formation, suggesting that the action of testosterone may primarily occur in non-ovarian tissues, which is the key site for the androgenic action that produces the PCOS phenotype (Caldwell et al., 2017).

#### 4.3.2 Obesity influence HPO axis via gut microbiota

##### 4.3.2.1 Abnormal gut microecology in obesity and PCOS individuals

Obesity is known to disrupt the gut microbiota, which plays a pivotal role in regulating metabolism, immunity, and endocrine functions. Dysbiosis, or imbalanced gut microbiota, is increasingly recognized as a contributing factor in metabolic and reproductive disorders, including obesity, T2DM, and PCOS (Winter and Bäumler, 2023).

Studies have shown that obesity is characterized by reduced microbial diversity, with a lower ratio of Bacteroidetes to Firmicutes compared to healthy controls (Ley et al., 2006). In obese individuals, the gut microbiota undergoes compositional changes, including an increase in Actinobacteria and a decrease in Bacteroidetes, which correlate with systemic inflammation, IR, and disrupted endocrine signaling (Chambers et al., 2019; Greenhill, 2015; Turnbaugh et al., 2009). Additionally, metagenomic sequencing of fecal nucleic acids has also revealed that patients with irregular menstrual cycles exhibit higher levels of *prevotella* and lower levels of *clostridiales*, *ruminococcus*, and *lachnospiraceae* (butyrate-producing bacteria) compared to those with regular cycles, suggesting a potential link between gut microbiota and female reproductive health (Sasaki et al., 2019).

Moreover, HFD impairs gut barrier function, leading to endotoxemia and chronic inflammation, IR, hyperandrogenism, and ovarian dysfunction—key features of PCOS (Tremellen and Pearce, 2012). Alterations in gut microbial diversity and composition are also prominent features in patients with PCOS and rodent models of PCOS (Li et al., 2024; Qi X. et al., 2019). For instance, specific microbial signatures have been identified in PCOS patients and animal models, such as an increase in *Bacteroides vulgatus* and a reduction in *Odoribacter* in the feces of hyperandrogenic PCOS mice (Qi X. et al., 2019; Yang et al., 2024). This suggests that gut microbiota dysbiosis may directly contribute to the metabolic and reproductive disturbances observed in obesity and PCOS. Furthermore, hyperandrogenic PCOS patients also exhibit decreased microbial richness, characterized by an increase in genera such as *bifidobacterium*, unclassified *enterobacteriaceae*, *streptococcus*, *saccharomycetaceae*, *enterococcus*, and the *eubacterium nodatum* (Li et al., 2024).

**FIGURE 4:**
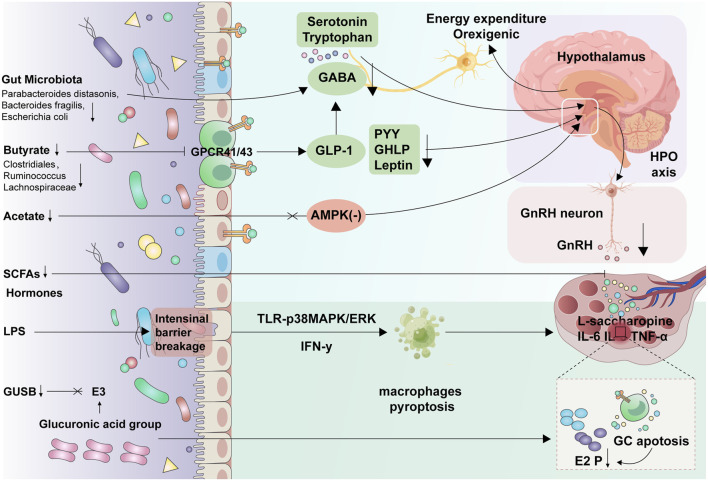
Gut microbiota-brain axis and gut microbiota-ovary axis The gut microbiota reduces the production of SCFAs, such as acetate and butyrate, which affects the production of gut peptides like GLP-1, PYY, GHRP, and Leptin, as well as neurotransmitters like serotonin, GABA, and tryptophan, thereby weakening the hypothalamic satiety effect. Moreover, dysregulation of microbial metabolism increases intestinal permeability and LPS levels, leading to hypothalamic insulin and leptin resistance, which in turn affects appetite and metabolism. In PCOS mice, LPS induces ovarian macrophage pyroptosis via IFN-γ and TLR-p38MAPK/ERK, affecting estrogen synthesis in GC, increasing the production of inflammatory factors like IL-6 and TNF-α, and leading to abnormal oocyte development. SCFA: short-chain fatty acids, GABA: gamma-aminobutyric acid, PCOS: polycystic ovary syndrome, LPS: lipopolysaccharides, Growth Hormone-Releasing Peptide.

Current research suggests that the gut microecosystem may influence physiological and pathological processes through several mechanisms: a) Hormonal Regulation: The gut microbiota produce bioenzymes that assist in nutrient digestion and metabolite synthesis and may be involved in hormone degradation and modification, affecting host metabolism and reproduction (Ervin et al., 2019); b) Endocrine Signaling: The gastrointestinal tract produces hormones like ghrelin, NPY, growth hormone-releasing peptide, GLP-1, and gastric inhibitory polypeptide (GIP), which act on organs such as the hypothalamus, pituitary, adrenal glands, and ovaries, thus regulating metabolism and the HPO axis (Izzi-Engbeaya and Dhillo, 2022); c) Gut-Derived Metabolites: Metabolites such as short-chain fatty acids (SCFAs), branched-chain amino acids (BCAA), LPS, and bile acids (BA) have diverse biological activities, regulating metabolism, immunity, inflammation, and endocrine functions (Figure 4; Joyce and Clarke, 2024).

##### 4.3.2.2 Gut microbiota and sex hormones

The impact of obesity and hormonal imbalances on gut microbiota has become an important area of research, particularly in relation to reproductive health. One key factor influencing gut microbial composition is the presence of androgens. For instance, studies on female rats treated with dehydroepiandrosterone (DHEA) have demonstrated changes in gut microbiota composition, such as a decrease in *Bacteroides* and an increase in anaerobes and *Clostridium*. Transplanting feces from DHEA-treated rats into pseudo-germ-free recipient rats induced metabolic and reproductive dysfunction phenotypes of PCOS, suggesting that androgen exposure may influence metabolic and reproductive functions by altering gut microbial composition (Kawai et al., 2021).

In addition, maternal exposure to androgens during pregnancy has been shown to impact the microbiota of offspring. One study revealed that prenatal androgen exposure increased the abundance of bacteria associated with steroid hormone synthesis, such as *nocardiaceae* and *clostridiaceae*, while decreasing the abundance of *akkermansia*, *bacteroides*, *lactobacillus*, and *clostridium* (Sherman et al., 2018). Similarly, rats exposed to androgens early in life also exhibited metabolic dysfunction and decreased gut microbial diversity in adulthood, with significant increases in the abundance of *firmicutes* and *Bacteroidetes* (Kawai et al., 2021).

Although the exact mechanisms remain unclear, sex hormones, including estrogens and androgens, likely influence gut microbiota through both direct and indirect actions. For example, sex hormones such as E2 and P may regulate bacterial metabolism directly through their receptors on gut bacteria, including estrogen receptor beta (ERβ) (Chen and Madak-Erdogan, 2016). Moreover, these hormones can serve as growth factors for certain anaerobic bacteria, promoting their proliferation (Hussain et al., 2021). Additionally, sex hormones may influence gut microbiota metabolism by altering substrate availability, a process mediated by changes in bacterial β-glucuronidase (GUSB) activity. Many gut bacteria produce GUSB enzymes that catalyze the release of glucuronic acid from host-derived substrates, such as sex hormones, which gut bacteria then use as a carbon source to promote their growth (Walsh et al., 2020). Furthermore, sex hormones may indirectly affect the gut microbiota by altering the gut microenvironment or modulating immune function through receptors in gut cells (Coquoz et al., 2022; Nie et al., 2018).

Sex hormones can influence the characteristics of gut microbiota, which in turn modulate hormonal homeostasis in the host. For example, the gut microbiota contains genes encoding estrogen-metabolizing enzymes, defined as the “estrobolome,” of which GUSB is considered a member (Ervin et al., 2019). During estrogen metabolism, E2 is first inactivated in the liver and conjugated with glucuronic acid by UDP-glucuronosyltransferase (UGT), allowing its excretion into the intestine via bile. Intestinal microbiota then deconjugate glucuronic acid using GUSB, leading to reabsorption of estrogen into the enterohepatic circulation, thereby increasing overall estrogen levels in the body (Sher and Rahman, 2000). Disruption of this process due to gut microbiota dysfunction, such as reduced bacterial GUSB activity, can alter hormone dissociation processes, leading to changes in circulating hormone levels, potentially contributing to the development of obesity, metabolic syndrome, CVD, and cognitive decline. Conversely, an increase in the abundance of GUSB-producing bacteria may induce pathological conditions of estrogen excess, leading to elevated circulating free estrogen levels and contributing to the development of diseases such as endometriosis and other estrogen-related cancers (Wei et al., 2023). Although there is no direct evidence on whether female gut microbiota can regulate testosterone levels in the body, studies in male mice have shown that non-glucuronidated DHT is 70 times higher in feces than in serum, indicating that the gut microbiota is involved in deglucuronidation (Kawai et al., 2021). In contrast, high levels of glucuronidated testosterone and DHT were detected in the distal colon of germ-free mice, while free DHT levels were very low. This study suggests that gut microbiota may also regulate host serum androgen levels through the process of deglucuronidation. The effect of gut microbiota on androgen levels has also been demonstrated through fecal microbiota transplantation (FMT) experiments, such as transplanting gut microbiota from female rats into adult male rats, which reduced testosterone levels (Feješ et al., 2024), while transplanting gut microbiota from PCOS patients or *B. vulgatus* into germ-free female mice increased testosterone levels, induced IR, and disrupted estrous cycles, resulting in PCOS-like phenotypes in the recipient mice (Qi X. et al., 2019). Previous studies have shown that FMT is insufficient to reduce the BMI or fat mass of obese patients; however, it has beneficial effects on glucose homeostasis, insulin sensitivity, lipid profile, and metabolism (Guzzardi et al., 2023).

##### 4.3.2.3 The gut-microbiota-brain axis

The “gut-microbiota-brain axis” refers to the bidirectional communication between the brain and gut microbiota through neuronal pathways, the immune system, and neurotransmitter-mediated signaling networks. This axis is fundamental in maintaining homeostasis in the central nervous system and the gastrointestinal system (Morais et al., 2021). Certain gut microbes produce SCFAs such as acetate, propionate, and butyrate, which activate G protein-coupled receptors (GPCRs) in enteroendocrine cells (EECs), to release appetite-regulating hormones such as GLP-1, PYY, growth hormone-releasing peptide (GHRP), and leptin, which then act on the hypothalamus to influence feeding behavior, satiety, and energy balance (Samuel et al., 2008; Tolhurst et al., 2012). Additionally, gut-derived SCFAs, such as acetate, can directly influence the hypothalamus by modulating the activity of neuropeptides like NPY and AgRP through the AMPK pathway, which is involved in suppressing appetite (Frost et al., 2014). Gut microbes also produce various neurotransmitters, such as serotonin, GABA, and tryptophan, which can act on the hypothalamus to influence its neuroendocrine functions, thereby influencing appetite regulation. Lastly, alterations in gut microbiota can increase gut permeability, leading to elevated levels of LPS in the bloodstream. This promotes systemic and hypothalamic inflammation, mediating insulin and LR and consequently affecting feeding behavior and metabolism.

Beyond appetite regulation, the gut-brain axis also plays a role in reproductive endocrinology, particularly in conditions like obesity and PP. A clinical study found significant differences in the gut microbiota of girls with CPP compared to healthy controls, with CPP patients showing increased abundances of *alistipes*, *klebsiella*, and *sutterella* (Li et al., 2021). These gut bacteria may influence neuroactivity by modulating pathways such as NO synthesis, which can stimulate GnRH secretion, a key regulator of puberty onset (Ceccatelli et al., 1993). Elevated NO might also promote IR, linking obesity and CPP (Sansbury and Hill, 2014). Acetate synthesis and NO synthesis were higher in CPP patients (Li et al., 2021). In animal models, HFD advanced puberty onset in female mice while also increasing serum E2, leptin, deoxycholic acid (DCA), and GnRH levels (Bo et al., 2022). Supplementation with SCFAs, such as acetate, propionate and butyrate, has been shown to reverse early puberty and restore normal GnRH levels in these models (Wang et al., 2022). Furthermore, transplantation of HFD microbiota into germ-free mice resulted in early puberty, suggesting that gut microbiota directly influence GnRH production through hypothalamic signaling (Bo et al., 2022).

In PCOS, alterations in gut microbiota composition and SCFA production may also contribute to hormonal imbalances. A clinical study of PCOS patients found an increase in GABA-producing gut bacteria, including *bacteroides distasonis, bacteroides fragilis,* and *escherichia coli,* which were significantly positively correlated with serum LH levels and the LH/FSH ratio (Liang et al., 2021). GABA, a neurotransmitter, is believed to promote GnRH and LH secretion, which may contribute to the reproductive features associated with PCOS (Silva et al., 2019). Additionally, SCFA levels are often reduced in PCOS patients; however, supplementation with probiotic *Lactobacillus* bifidus V9 has been shown to restore SCFA production, balance hormone levels, and reduce LH/FSH ratios, suggesting a role for SCFAs in gut-brain axis-mediated regulation of the central nervous system (Zhang et al., 2019).

The role of GLP-1 in mediating the effects of gut microbiota on reproductive health has also gained attention. As an important gut hormone (Huang X. et al., 2024), GLP-1 production is also influenced by gut microbiota. For example, studies have found that antibiotic-induced reduction of Firmicutes and Bacteroidetes in the gut of mice significantly increased serum GLP-1 levels and GLP-1 expression in gut endocrine L cells, improving IR in diet-induced obese mice (Hwang et al., 2015). Similarly, dietary fiber intake can increase the production of SCFAs by gut microbiota, which subsequently stimulates GLP-1 secretion by acting on GPR41/FFAR3 and GPR43/FFAR2 on gut endocrine L cells, thereby improving obesity and IR (Samuel et al., 2008; Tolhurst et al., 2012; Nøhr et al., 2013). GLP-1 has been shown to influence LH synthesis and GnRH neuronal activity, enhancing reproductive functions in animal models (Farkas et al., 2016; Outeiriño-Iglesias et al., 2015). These studies suggest the potential role of GLP-1 in the pathogenesis of PCOS. Due to the efficacy of GLP-1 agonists in improving metabolic and reproductive endocrine dysfunction in PCOS, they are considered promising therapeutic agents for this condition (Figure 4; Babar et al., 2023).

##### 4.3.2.4 The gut-microbiota-ovary axis

Emerging evidence supports the existence of a direct “gut-microbiota-ovary axis,” where gut microbiota can influence ovarian function. Alterations in gut microbiota composition have been shown to affect ovarian function, as demonstrated by experiments in female mice where transplantation of gut microbiota from young mice into older mice led to improved follicular development and enhanced ovarian function (Xu et al., 2022). Similarly, supplementation with an appropriate amount of *lycium barbarum* polysaccharide promoted follicular development by increasing the beneficial gut microbiota such as *faecalibaculum*, *bilophila*, and *anaerofustis* in female mice (Zheng et al., 2023).

Differences in gut microbiota composition have also been linked to fertility in different animal breeds. For instance, Meishan sows with higher fertility exhibited larger ovarian weights, better follicular development, more functional follicles, fewer atretic follicles, and less granulosa cell apoptosis compared to Landrace × Yorkshire (L × Y) sows with lower fertility. These breeds also exhibited different gut microbiota and metabolomic characteristics, with Meishan sows displaying higher gut microbial α-diversity, stronger carbohydrate metabolism in the feces, and higher levels of SCFAs (Xu et al., 2023). Further research revealed that SCFAs produced by these gut bacteria protect ovarian granulosa cells from apoptosis and enhance ovarian hormone production (Xu et al., 2023). Butyrate, in particular, enhanced E2 and P production in porcine granulosa cells by activating GPCRs (Lu et al., 2017). Moreover, SCFAs, such as butyrate, were found to mitigate ovarian inflammation in obese PCOS mice, further supporting the role of SCFAs in regulating ovarian function (Liu K. et al., 2023).

Conversely, gut microbiota, particularly an overabundance of endotoxin-producing bacteria such as Desulfovibrio, can lead to elevated circulating LPS levels, which induce inflammation in the ovaries, increase intestinal permeability, and impair estrogen synthesis, thereby contributing to reproductive dysfunction in obesity and PCOS (Taylor and Terranova, 1995; Tremellen et al., 2015). In HFD mice, impaired intestinal barrier results in the significant accumulation of L-saccharopine in the feces, serum, and ovaries, leading to mitochondrial dysfunction that subsequently impacts oocyte quality and reduces estrogen production (Wen et al., 2024). LPS also stimulates granulosa cells to produce pro-inflammatory factors such as IL-6, IL-8, and TNF-α through non-innate immune pathways mediated by TLR-p38MAPK/ERK, influencing follicular health and increasing rates of meiotic arrest in oocytes (Bromfield and Sheldon, 2011). In DHEA-induced PCOS mice, an increase in gut Gram-negative bacteria such as *desulfovibrio* and *burkholderia* led to high LPS levels, which induced pyroptosis in ovarian macrophages mediated by IFN-γ, resulting in impaired estrogen synthesis and increased apoptosis of granulosa cells (Figure 4; Huang et al., 2022). These findings suggest that local ovarian inflammation induced by elevated LPS levels due to gut microbiota dysbiosis and increased gut permeability may be a significant contributor to obesity-related ovarian dysfunction.

Gut microbiota provides a crucial link between obesity and reproductive health by influencing both metabolic and hormonal pathways. Dysbiosis in obesity leads to systemic inflammation, IR, and altered sex hormone metabolism, all of which contribute to the development of reproductive disorders such as PCOS. As discussed, gut microbiota directly affects the HPO axis, modulate the production of reproductive hormones, and impact ovarian health through SCFAs, endotoxins, and other metabolites. Furthermore, these microbial changes are not merely passive bystanders but actively contribute to the pathogenesis of obesity-related reproductive dysfunctions.

## 5 Obesity interventions for the treatment of female reproductive endocrine disorders

### 5.1 Dietary and behavioral interventions

The “International Evidence-Based Guideline for the Assessment and Management of PCOS” emphasizes the integration of a healthy lifestyle into comprehensive PCOS management to control weight gain (Teede et al., 2018). Dietary strategies, such as the Mediterranean diet (MD), emphasize the consumption of phytonutrient-dense foods, including olive oil, non-starchy vegetables, legumes, nuts, unsaturated fats, and low-fat dairy products (Kiani et al., 2022). The MD has been shown to enhance success rates for IVF, improve clinical pregnancy rates, and better live birth outcomes, alongside a reduction in gestational hypertension and diabetes (Yang et al., 2023). SCFAs, beneficial metabolites produced by gut microbiota during the fermentation of undigested dietary fiber, can be generated from the oligosaccharides and resistant starches present in the MD, inhibiting gastric emptying, increasing satiety, stimulating GLP-1 release, enhancing insulin sensitivity, and supporting weight loss (Barrea et al., 2019). The Dietary Approaches to Stop Hypertension (DASH) diet, which prioritizes the intake of vegetables, fruits, whole grains, low-fat or non-fat dairy products, fish, poultry, nuts, and seeds while limiting sodium, red meat, processed meats, and sugary beverages, has been associated with more significant weight loss in overweight or obese individuals compared to other dietary patterns, with an average weight reduction of 3.08 kg after 1 year (Ge et al., 2020). The DASH diet effectively lowers fasting insulin levels, which are closely linked to lipid accumulation and reduced insulin clearance associated with obesity (Koh et al., 2022). Several randomized controlled clinical trials have demonstrated that the DASH diet can improve metabolic disorders, HA conditions, and biomarkers of oxidative stress in women with PCOS, highlighting its potential as an effective treatment modality (Barrea et al., 2023). The ketogenic diet (KD), characterized by high fat, moderate protein, and low carbohydrate intake (Barrea et al., 2023), has been shown to reduce postprandial insulin secretion and improve IR through weight loss. It activates AMPK and SIRT1, improving glucose homeostasis and insulin sensitivity in PCOS (Paoli et al., 2020). Additionally, decanoic acid (DA), a component of medium-chain triglyceride diet (MCT diet), has been found to lower serum free testosterone levels, reduce fasting insulin levels, and restore estrous cycles in letrozole-induced PCOS rat models (Lee et al., 2016). Beyond these reproductive benefits, the metabolic improvements are linked to the modulation of gut hormones, as evidenced by research showing that MCTs/their components enhance glucose metabolism through GLP-1 secretion in both animal models and cell lines (Nonaka et al., 2022). Our unpublished data suggest that the MCT diet positively affects the metabolic and reproductive outcomes of women with PCOS and DHT-induced PCOS mice, and that DA can affect the steroidogenic function of granulosa cells by directly acting on PPARγ. Several randomized controlled trials have evaluated the effects of probiotics and synbiotics in women with PCOS, showing improvements in IR, reductions in androgen levels, and positive changes in lipid profiles. By supplementing with prebiotics (such as fructooligosaccharides and inulin) and probiotics (such as bifidobacteria and lactobacilli) to directly increase the number of beneficial bacteria, it has shown potential in multiple clinical trials to improve metabolic and endocrine indicators in PCOS (Martinez Guevara et al., 2024). In addition to these dietary patterns, single dietary approaches such as high-protein, low-calorie, and low-glycemic-index diets have also demonstrated efficacy in weight management (Juhász et al., 2024).

Behavioral interventions particularly physical exercise, have shown potential in improving fertility and reducing pregnancy complications by restoring ovulation (Schenkelaars et al., 2021). However, a systematic review and meta-analysis by the U.S. Preventive Services Task Force, which included 89 trials on behavior-based weight loss and weight maintenance, indicated that behavioral interventions alone may not produce clinically significant weight control, although they can improve quality of life (LeBlanc et al., 2018). Previous studies suggest that while exercise can reduce weight and improve IR in PCOS patients, dietary interventions often yield more substantial results (Jungari et al., 2023). Compared with dietary interventions, higher adherence and individualization are more challenging to achieve with behavioral interventions, which may explain the suboptimal effectiveness of behavioral interventions. However, we place more emphasis on combining dietary and behavioral interventions.

### 5.2 Therapeutics targeted gut microbiota

At present, therapeutic approaches targeting intestinal flora and its metabolites, such as probiotics, prebiotics, synbiotics and FMT, have made significant progress in the treatment of obesity and related metabolic diseases. These methods aim to influence host metabolism and improve disease states by modulating the composition and function of gut microbiota (Gao et al., 2024). Probiotics are live microbes that benefit the host when consumed in sufficient quantities (Ku et al., 2024). Common probiotics, such as lactic acid bacteria and bifidobacteria,can regulate the composition of intestinal flora, enhance intestinal barrier function, and produce beneficial metabolites such as SCFAs, thereby affecting host energy metabolism, inflammatory response and improving IR (Borgonovi et al., 2022; Lin et al., 2022). Prebiotic is a food component that can not be digested and absorbed by the host but can selectively promote the growth and activity of beneficial microorganisms in the intestinal tract. Common prebiotics include inulin, fructooligosaccharides, resistant starch, and galactooligosaccharides. By promoting the proliferation of beneficial bacteria such as Bifidobacterium and *Lactobacillus*, prebiotics can improve gut microbiota composition, reduce energy intake, reduce oxidative stress, inflammatory responses, and improve insulin resistance, thereby reducing the risk of CVD, diabetes and hypertension and indirectly improve metabolic health (Borgonovi et al., 2022; Jacquier et al., 2024). Synbiotics are a combination of probiotics and prebiotics that work together to enhance the survival and activity of probiotics in the gut, resulting in more significant health benefits (Al-Habsi et al., 2024). FMT is the transfer of a healthy donor’s fecal microbiota into a patient’s gut to restore a healthy intestinal microecosystem. It can correct intestinal flora imbalance, restore microbial diversity, increase SCFA production, improve intestinal barrier function, and reduce bacterial translocation and systemic inflammation, thereby having a positive impact on metabolic health (Hatton et al., 2020).

The research on the treatments targeting intestinal microecology in female reproductive endocrine related diseases are still in their infancy, mainly focusing on the study of PCOS. Systematic reviews and meta-analyses suggest that probiotic supplements may improve insulin resistance, blood glucose and lipid levels in PCOS patients (Martinez Guevara et al., 2024; Angoorani et al., 2023). Probiotics such as *Lactobacillus* reuteri, for example, have been found to reduce weight, reduce IR and improve fertility in PCOS patients (Basnet et al., 2024). Experimental studies have found that supplement of *E. coli* Nissle 1917 (EcN) can improve mitochondrial damage of granular cells in PCOS mice by promoting the production of intestinal immune factor IL-22 (Luo et al., 2023). Probiotic supplementation can reduce circulating androgen levels and improve hyperandrogenemia in PCOS individuals. It also increases intestinal microbial diversity, inhibits the growth of harmful bacteria, thereby reducing inflammatory levels in PCOS patients by enhancing intestinal barrier function and decreasing the production of pro-inflammatory cytokines (Artyomenko et al., 2023; Zhang H. et al., 2023). As a prebiotic, inulin regulates gut microbiota to significantly improve hyperandrogenemia and glucose-lipid metabolism in PCOS patients and model mice (Geng et al., 2025). It enhances the production of butyrate by gut bacteria, thereby improving insulin sensitivity in PCOS patients (Geng et al., 2025). Synbiotics may be more effective than using probiotics or prebiotics alone in improving clinical symptoms and metabolic indicators of PCOS (Martinez Guevara et al., 2024). In addition, transferring fecal microbiota from PCOS patients to germ-free mice leads to metabolic disorders and ovarian dysfunction in recipient mice, strongly demonstrating the direct role of gut microbiota in PCOS pathogenesis. Conversely, transplanting fecal microbiota from healthy donors to PCOS model animals shows promise in improving metabolic abnormalities and reproductive endocrine functions, indicating a potent theraputic value of FMT in treatment of PCOS (Huang F. et al., 2024).

### 5.3 Pharmacotherapy

The FDA has approved several long-term weight-loss medications, including orlistat, phentermine/topiramate extended-release, naltrexone/bupropion extended-release, liraglutide, and semaglutide. Clinical studies have demonstrated that these pharmacotherapies can achieve weight reductions ranging from 3.07 kg to 9.77 kg (Singh and Singh, 2020). Orlistat, a gastrointestinal lipase inhibitor, has been shown to improve ovulation rates in PCOS patients when used in combination with oral contraceptives compared to oral contraceptives alone (Legro et al., 2015). Both liraglutide and semaglutide, GLP-1 receptor agonists, are effective for weight management and have potential as treatments for obese patients with PCOS (Austregésilo de Athayde De Hollanda Morais et al., 2024). GLP-1 agonists exhibits anti-inflammatory and anti-fibrotic properties in the ovaries and endometrium, increases insulin sensitivity, delays gastric emptying, and enhances satiety, while also improving menstrual regularity in PCOS, reducing serum free testosterone levels, and decreasing ovarian stromal volume (Nylander et al., 2017). Metformin, a well-established insulin sensitizer with a long-term safety profile, is also employed for weight management. Its efficacy is not only observed in short-term studies (showing a 2%–7% reduction) but is particularly notable for long-term maintenance. Evidence from the Diabetes Prevention Program Outcomes Study (DPPOS) demonstrated that individuals on metformin who achieved initial weight loss maintained a mean loss of 6.2% over 15 years (Apolzan et al., 2019; Day et al., 2019). Evidence-based guidelines recommend metformin for weight loss and the management of endocrine-metabolic disorders (Legro et al., 2013). Metformin can significantly and sustainably alter gut microbiota composition, such as increasing *E. coli* and R. torques while reducing I. bartlettii and R. intestinalis. Additionally, metformin significantly changed 62 microbial functional pathways, including acetate production and glucose metabolism, and increased serum SCFAs (butyrate, acetate, valerate) (Mueller et al., 2021). Pharmacotherapy should be re-evaluated after 3–4 months, with discontinuation considered if weight loss is less than 4%–5%.

### 5.4 Surgical treatment

Bariatric surgery, including Roux-en-Y Gastric Bypass (RYGB), Sleeve Gastrectomy (SG), adjustable gastric banding (AGB), and biliopancreatic diversion (BPD), is effective not only in achieving weight loss but also in directly improving reproductive health in obese women (Ciangura et al., 2019). SG and RYGB are the most common procedures, accounting for 61% and 17% of all bariatric surgeries, respectively (Arterburn et al., 2020). Among bariatric surgery patients, women of childbearing age represent approximately 49%, highlighting the importance of weight control in this population (Ciangura et al., 2019). Compared to obese women who did not undergo surgery, those who did show improved pregnancy outcomes, including reduced risks of gestational diabetes (Brüning et al., 2000; Kawai et al., 2021), preeclampsia, gestational hypertension, and better neonatal outcomes, such as lower incidences of low birth weight and macrosomia (Maggard et al., 2008). Bariatric surgery is highly effective in promoting weight loss, restoring menstrual cycles, reducing serum androgen levels, and providing metabolic benefits (Escobar-Morreale et al., 2005; Singh et al., 2020). For instance, a prospective study of Indian women with PCOS demonstrated a 63% excess weight loss at 1 year, accompanied by a significant drop in serum testosterone and the restoration of normal menstrual cycles in all patients within 3 months, thereby validating its comprehensive efficacy (Singh et al., 2020). A cross-sectional study of 515 obese women (BMI 42.2 ± 7.5 kg/m^2^) who underwent gastric banding or gastric bypass surgery, with an average weight loss of 35.3 ± 17.9 kg, revealed a reduction in the proportion of women with irregular menstrual cycles (>35 days) from 38% to 25% (Różańska-Walędziak et al., 2020). Typically, normal menstrual cycles resume 3 months post-surgery (Bilenka et al., 1995), with weight stabilizing within 1–2 years. A comprehensive review demonstrates that this metabolic transformation is highly beneficial for female reproductive health, leading to the normalization of reproductive hormones, improved fertility, and a decreased risk of miscarriage (Merhi, 2009). The 2019 clinical practice guidelines on perioperative nutrition, metabolism, and non-surgical support for bariatric surgery patients recommend postponing pregnancy until 12–18 months after surgery to prevent nutritional deficiencies in the fetus due to rapid maternal weight loss (Mechanick et al., 2019). Bariatric surgery may alter ghrelin release levels, a key regulator of both obesity and reproduction, due to structural changes in the stomach. Post-surgery, ghrelin levels remain reduced for over a year, further influencing metabolism and reproductive health (Jacobsen et al., 2012; Sundbom et al., 2007).

### 5.5 Traditional and alternative medicine therapies

Emerging research indicates that traditional Chinese medicine (TCM), along with practices such as tai chi and yoga, can effectively address reproductive endocrine disorders (Ye et al., 2022). Polysaccharides, important active compounds in many TCMs, are recognized for their prebiotic activity and contributions to disease prevention and treatment. Polysaccharides derived from *Cordyceps sinensis* and *Ganoderma lucidum* have been shown to inhibit weight gain and fat accumulation in HFD obese mice (Kawai et al., 2021). Additionally, Polysaccharides from *lycium barbarum* and *schisandra* have been observed to increase SCFA content and modulate the expression of inflammatory factors, thereby exerting anti-inflammatory effects (Chen et al., 2019; Qi Y. et al., 2019). Moreover, *Codonopsis pilosula* polysaccharides exhibit antioxidant properties through the activation of the Nrf2 signaling pathway, leading to improved IR in a high-fat, high-sucrose diet obese mice model (Zhang et al., 2020). Furthermore, polysaccharides from other traditional Chinese medicines, including *Codonopsis*, *Ginseng*, *Cistanche*, and *Ophiopogon*, have been shown to restore gut microbiota balance and elevate SCFA levels, contributing to anti-inflammatory and anti-obesity effects (Cao et al., 2022; Fu et al., 2020; Li S. et al., 2019; Wang et al., 2019). Similarly, resveratrol from *Polygonum cuspidatum*, as well as polysaccharides and saponins from *Gynostemma pentaphyllum*, also display prebiotic properties (Kawai et al., 2021). In addition to influencing the gut microbiota, TCM may also impact the gut structure to exert control over BW Studies have demonstrated that puerarin, a compound found in Pueraria lobata, can inhibit the activity of dorsal motor nucleus of the vagus (DMV) neurons by binding to the GABA type A receptor subunit alpha1 (GABRA1). Downregulation of Ezrin, CDC42, Eps8, and Villin 1, which are crucial for maintaining intestinal microvilli length. Consequently, leading to a reduction in its length, which in turn suppresses fat absorption and controls weight (Lyu et al., 2024). Berberine has been demonstrated to improve insulin sensitivity and IR. Evidence from a dehydroepiandrosterone-induced PCOS rat model indicates that its insulin-sensitizing effects are concomitant with the downregulation of key pro-inflammatory markers (TLR4, NF-κB, TNF-α) and apoptosis-related proteins (caspase-3), suggesting that berberine alleviates IR, at least in part, through anti-inflammatory and anti-apoptotic mechanisms (Shen et al., 2021), Furthermore, berberine is capable of regulating dyslipidemia and reducing androgen levels, as well as the LH/FSH ratio (Xie et al., 2019). Additionally, berberine facilitates ovulation by modulating the expression of LHCGR and CYP19A1 genes in granulosa cells, which are crucial for the development of female gonads. Specifically, in PCOS, berberine can enhance ER by upregulating the expression of lysophosphatidic acid receptor 3 (LPAR3) and integrin αvβ3 proteins in endometrial tissue (Wang et al., 2021). These studies suggest that TCM often plays a systemic regulatory role. Systemic intervention methods are the current direction of treatment research for chronic diseases such as obesity. Therefore, TCM still has a vast space for further research (Lyu et al., 2024). The advantages and limitations is shown in Table 2.

**TABLE 2:** The advantages and Limitations of each treatment.

Intervention category	Specific intervention	Advantages	Limitations
Dietary Interventions	MD (Yang et al., 2023; Barrea et al., 2019)	Improves metabolism, enhances IVF success rates and live birth outcomes, reduces gestational complications, and is rich in prebiotics, beneficial for gut microbiota	Requires long-term adherence
	DASH Diet (Ge et al., 2020; Koh et al., 2022)	Improves metabolic disorders and oxidative stress in PCOS, and results in significant weight loss	Strict limits on sodium and red/processed meats can be monotonous, and adherence can be challenging
	KD (Barrea et al., 2023; Paoli et al., 2020)	Improving insulin resistance and reduce postprandial insulin secretion, improving glucose homeostasis and insulin sensitivity	Poor long-term adherence; Constipation, headaches; limited long-term safety data; low in fiber and prebiotics
	MCT Diet (Lee et al., 2016; Nonaka et al., 2022; Martinez Guevara et al., 2024)	Directly modulates granulosa cell function (via PPARγ); lowers testosterone, restores estrous cycles; improves glucose metabolism	MCT oil can cause gastrointestinal distress
Microbiome-Targeted Therapy	Probiotics/Synbiotics (Ku et al., 2024; Borgonovi et al., 2022; Lin et al., 2022; Basnet et al., 2024; Luo et al., 2023; Artyomenko et al., 2023; Zhang et al., 2023b)	Directly supplements beneficial bacteria; improves IR, androgen levels, and lipid profiles	Effects are strain-specific; require continuous intake, effects may not persist after cessation; mixed research results
	Prebiotics (FOS, Inulin) (Borgonovi et al., 2022; Jacquier et al., 2024; Geng et al., 2025)	Stimulates the growth of beneficial gut bacteria and improves metabolic and endocrine markers in PCOS.	Can cause gastrointestinal side effects (bloating, flatulence), especially in individuals with IBS or FODMAP intolerance
	FMT (Hatton et al., 2020; Huang et al., 2024b)	Corrects endocrine and metabolic disorders via improving intestinal microecological imbalance, insulin sensitivity, inflammatory response, and provide a new non-invasive treatment approach	The transplantation effect is variable; the clinical safety need to be further verified by large-scale studies; the long-term maintenance effect is still unclear; potential infection and ethical issues are existed
Pharmacotherapy	Metformin (Apolzan et al., 2019; Day et al., 2019)	Improves insulin sensitivity, well-established long-term safety profile; modulates gut microbiota and increases SCFAs	Modest weight loss efficacy (2%–7%); common gastrointestinal side effects (diarrhea, nausea)
	GLP-1 Agonists (e.g., Liraglutide, Semaglutide) (Austregésilo de Athayde De Hollanda Morais et al., 2024)	Highly effective for weight loss; improves menstrual regularity, reduces testosterone; has anti-inflammatory/anti-fibrotic properties on the ovaries	High cost; Gastrointestinal side effects (nausea, vomiting, diarrhea); requires injection (except oral semaglutide)
	Orlistat (Legro et al., 2015)	Non-systemically acting weight-loss drug; improves ovulation rates in PCOS when combined with oral contraceptives	Common side effects (oily stools, fecal urgency); Malabsorption of fat-soluble vitamins
Surgical Treatment	Bariatric Surgery (e.g., RYGB, SG) (Arterburn et al., 2020; Maggard et al., 2008; Escobar-Morreale et al., 2005; Singh et al., 2020)	Most effective and sustained weight loss; dramatically improves menstruation, fertility, androgen levels, and pregnancy outcomes	Surgical risks and complications; requires lifetime dietary modifications and nutritional supplementation
TCM	Polysaccharides (Chen et al., 2019; Qi et al., 2019b; Zhang et al., 2020)	Systemic regulation via prebiotic effects offers anti-inflammatory, antioxidant, and metabolic benefits	Complex mechanisms; Clinical evidence remains insufficient
	Berberine (Shen et al., 2021; Xie et al., 2019; Wang et al., 2021)	Improves insulin sensitivity, dyslipidemia, and androgen levels; facilitates ovulation	Lower quality clinical evidence compared to conventional pharmaceuticals; potential for drug interactions; gastrointestinal discomfort
	Puerarin (Lyu et al., 2024)	Systemic regulation, mediated by mechanisms involving the vagus nerve, inhibits fat absorption	Lower quality clinical evidence

MD: mediterranean diet, DASH: dietary approaches to stop hypertension, KD: ketogenic diet, MCT: Medium-Chain Triglyceride, FMT: fecal microbiota transplantation, FOS: fructooligosaccharides, IBS: irritable bowel syndrome, FODMAP: fermentable oligosaccharides, Disaccharides, Monosaccharides and Polyols, SCFAs: Short-Chain Fatty Acids, GLP-1: Glucagon-like Peptide-1, RYGB: Roux-en-Y, gastric bypass, SG: sleeve gastrectomy, FMT: fecal microbiota transplantation, TCM: traditional chinese medicine, IVF: in vitro fertilization, PCOS: polycystic ovary syndrome, PPARγ: Peroxisome Proliferator-Activated Receptor Gamma, IR: insulin resistance.

## 6 Conclusion and future directions

The intricate relationship between obesity and female reproductive and endocrine functions has emerged as a critical area of contemporary biomedical research, driven by the escalating global prevalence of obesity (Yong et al., 2023). This interdisciplinary field recognizes that obesity is not merely a metabolic disorder but a significant contributor to a complex array of hormonal imbalances and reproductive dysfunctions, profoundly impacting women’s health and fertility. Advances in understanding the underlying mechanisms have highlighted the multifaceted nature of this interaction, involving hormonal alterations, chronic inflammation, metabolic perturbations, and disruptions along the hypothalamic-pituitary-ovarian (HPO) axis. In this review, we examined the co-regulatory mechanisms that link energy metabolism and female reproduction from an evolutionary perspective, highlighting the significance of these interconnected pathways in the pathogenesis of reproductive endocrine dysfunction. This insight suggests that targeted interventions addressing these mechanisms could simultaneously improve both metabolic health and reproductive endocrine function in obese women.

It is important to recognize that the negative impact of obesity on women’s reproductive health extends beyond disruptions in the HPO axis; it is also associated with various pregnancy complications and adverse outcomes (Schon et al., 2024), including higher rates of miscarriage and poorer outcomes in assisted reproductive technologies like *in vitro* fertilization (IVF) (Klenov and Jungheim, 2014). Additionally, reproductive endocrine dysfunction can be both a consequence of obesity and a contributor to metabolic disturbances. For instance, hyperandrogenism in women can induce obesity and IR, while postmenopausal hypoestrogenism and elevated FSH levels are closely associated with increased obesity risk in middle-aged and older women (Kim SM. et al., 2024; Zhu et al., 2023). Recent genetic studies have also begun to unravel the shared genetic bases between higher BMI and various female reproductive disorders, indicating a deeper, inherent connection beyond environmental factors. These studies have identified common risk loci and biological pathways, underscoring the evolutionary connection between metabolic processes and reproductive endocrine function, further illustrating the bidirectional relationship between these factors (Kwon and Cho, 2025; Shao et al., 2025).

Despite the insights gained, several critical scientific questions remain unresolved. Firstly, in the brain, neurons such as those in the hypothalamic GnRH and Kisspeptin systems are certainly not the only or primary targets for obesity and related endocrine and inflammatory factors. These factors may act on other brain regions or even higher-level brain regions, and indirectly affect the hypothalamic metabolism and reproductive regulation centers through complex neural circuits, thereby determining the impact of obesity on reproductive endocrinology. For instance, some recent studies also indicate that obesity can impair cognitive function and is associated with widespread changes in brain structure (Oliveras-Cañellas et al., 2023; Sakib et al., 2023). Furthermore, obesity is also associated with various mood disorders and behavioral abnormalities (Wang RZ. et al., 2024). Whether and how damage to multiple brain regions and functions affects the HPG axis requires further study. In terms of peripheral aspects, the molecular mechanisms by which obesity-related metabolic regulatory factors directly control gonadal germ cells and endocrine cells require more in-depth research. Through such studies, we may discover additional targets for improving reproductive endocrine-related diseases by improving obesity and metabolic conditions. For example, in our unpublished research, we found that PCOS mouse granulosa cells have PPARγ-dependent lipid metabolism disorders. The use of medium-chain triglyceride diets not only improved the lipid metabolism issues in PCOS mice but also, through the PPARγ pathway, altered the endocrine function of mouse granulosa cells. These findings also suggest that the connection between metabolism and reproduction involves not only certain neurotransmitters and endocrine factors but may also include important transcription factors related to metabolic regulation. Additionally, exploring sex-specific differences in metabolic responses and reproductive physiology is crucial. Historically, female physiology has been underrepresented in basic and clinical research (MacGregor et al., 2025). Addressing this gap will provide a more comprehensive understanding of how obesity uniquely affects women’s reproductive health compared to men.

Additionally, exploring more genetic and epigenetic co-regulatory mechanisms between metabolism and reproduction through advanced research methodologies, such as multi-omics research methods, will be imperative. In terms of treatment, further clinical research is essential to evaluate the effectiveness, safety, and specific strategies of obesity interventions, such as diet, exercise, medication, and surgery, in improving reproductive endocrine function and reproductive health. The integration of advanced technologies, such as artificial intelligence (AI) and computational modeling, holds significant potential for identifying individuals at risk, predicting treatment responses, and developing personalized management plans for obesity-related reproductive disorders. Such tools can help manage complex datasets generated from multi-omics research and clinical trials, leading to more efficient and effective interventions (Guan et al., 2023). The future of research in this area will undoubtedly be characterized by a holistic, interdisciplinary approach aimed at restoring optimal reproductive and endocrine health in obese women.

## References

[B1] Abbott D. H. Dumesic D. A. Eisner J. R. Colman R. J. Kemnitz J. W. (1998). Insights into the development of polycystic ovary syndrome (PCOS) from studies of prenatally androgenized female rhesus monkeys. Trends Endocrinol. Metab. 9 (2), 62–67. 10.1016/s1043-2760(98)00019-8 18406243

[B2] Acevedo N. Ding J. Smith G. D. (2007). Insulin signaling in mouse oocytes. Biol. Reprod. 77, 872–879. 10.1095/biolreprod.107.060152 17625112

[B3] Acton B. M. Jurisicova A. Jurisica I. Casper R. F. (2004). Alterations in mitochondrial membrane potential during preimplantation stages of mouse and human embryo development. Mol. Hum. Reprod. 10 (1), 23–32. 10.1093/molehr/gah004 14665703

[B4] Al-Habsi N. Al-Khalili M. Haque S. A. Elias M. Olqi N. A. Al Uraimi T. (2024). Health benefits of prebiotics, probiotics, synbiotics, and postbiotics. Nutrients 16, 3955. 10.3390/nu16223955 39599742 PMC11597603

[B5] Ampofo A. G. Boateng E. B. (2020). Beyond 2020: modelling obesity and diabetes prevalence. Diabetes Res. Clin. Pract. 167, 108362. 10.1016/j.diabres.2020.108362 32758618

[B6] Andrews Z. B. (2011). Central mechanisms involved in the orexigenic actions of ghrelin. Peptides 32, 2248–2255. 10.1016/j.peptides.2011.05.014 21619904

[B7] Angoorani P. Ejtahed H. S. Ettehad Marvasti F. Taghavi M. Mohammadpour Ahranjani B. Hasani-Ranjbar S. (2023). The effects of probiotics, prebiotics, and synbiotics on polycystic ovarian syndrome: an overview of systematic reviews. Front. Med. (Lausanne) 10, 1141355. 10.3389/fmed.2023.1141355 37359018 PMC10288857

[B8] Apolzan J. W. Venditti E. M. Edelstein S. L. Knowler W. C. Dabelea D. Boyko E. J. (2019). Long-term weight loss with metformin or lifestyle intervention in the diabetes prevention program outcomes study. Ann. Intern Med. 170 (10), 682–690. 10.7326/M18-1605 31009939 PMC6829283

[B9] Arbabi L. Li Q. Henry B. A. Clarke I. J. (2021). Glucagon-like peptide-1 control of GnRH secretion in female sheep. J. Endocrinol. 248, 325–335. 10.1530/JOE-20-0335 33446613

[B10] Arterburn D. E. Telem D. A. Kushner R. F. Courcoulas A. P. (2020). Benefits and risks of bariatric surgery in adults: a review. JAMA 324 (9), 879–887. 10.1001/jama.2020.12567 32870301

[B11] Artyomenko V. Nastradina N. Kozhukhar H. (2023). Changes in the microbiome in women with polycystic ovary syndrome. Reprod. Endocrinol., 30–35. 10.18370/2309-4117.2023.68.30-35

[B12] Austregésilo de Athayde De Hollanda Morais B. Martins Prizão V. de Moura de Souza M. Ximenes Mendes B. Rodrigues Defante M. L. Cosendey Martins O. (2024). The efficacy and safety of GLP-1 agonists in PCOS women living with obesity in promoting weight loss and hormonal regulation: a meta-analysis of randomized controlled trials. J. Diabetes Complicat. 38 (10), 108834. 10.1016/j.jdiacomp.2024.108834 39178623

[B13] Azziz R. Carmina E. Dewailly D. Diamanti-Kandarakis E. Escobar-Morreale H. F. Futterweit W. (2006). Positions statement: criteria for defining polycystic ovary syndrome as a predominantly hyperandrogenic syndrome: an androgen excess society guideline. J. Clin. Endocrinol. Metab. 91 (11), 4237–4245. 10.1210/jc.2006-0178 16940456

[B14] Babar A. Ali R. Zuhair V. (2023). Glucagon-like peptide 1 agonists: a ray of hope for the treatment of polycystic ovarian syndrome. J. Pak Med. Assoc. 73 (12), 2521–2522. 10.47391/JPMA.9666 38083956

[B15] Bahety D. Böke E. Rodríguez-Nuevo A. (2024). Mitochondrial morphology, distribution and activity during oocyte development. Trends Endocrinol. Metab. 35, 902–917. 10.1016/j.tem.2024.03.002 38599901

[B16] Balen A. H. Morley L. C. Misso M. Franks S. Legro R. S. Wijeyaratne C. N. (2016). The management of anovulatory infertility in women with polycystic ovary syndrome: an analysis of the evidence to support the development of global WHO guidance. Hum. Reprod. Update 22 (6), 687–708. 10.1093/humupd/dmw025 27511809

[B17] Barabás K. Barad Z. Dénes Á. Bhattarai J. P. Han S. K. Kiss E. (2018). The role of Interleukin-10 in mediating the effect of immune challenge on mouse gonadotropin-releasing hormone neurons *in vivo* . eNeuro 5 (5), 0211–18. 10.1523/ENEURO.0211-18.2018 30406179 PMC6220573

[B18] Barlampa D. Bompoula M. S. Bargiota A. Kalantaridou S. Mastorakos G. Valsamakis G. (2021). Hypothalamic inflammation as a potential pathophysiologic basis for the heterogeneity of clinical, hormonal, and metabolic presentation in PCOS. Nutrients 13 (2), 520. 10.3390/nu13020520 33562540 PMC7915850

[B19] Barrea L. Arnone A. Annunziata G. Muscogiuri G. Laudisio D. Salzano C. (2019). Adherence to the mediterranean diet, dietary patterns and body composition in women with polycystic ovary syndrome (PCOS). Nutrients 11 (10), 2278. 10.3390/nu11102278 31547562 PMC6836220

[B20] Barrea L. Verde L. Camajani E. Cernea S. Frias-Toral E. Lamabadusuriya D. (2023). Ketogenic diet as medical prescription in women with polycystic ovary syndrome (PCOS). Curr. Nutr. Rep. 12 (1), 56–64. 10.1007/s13668-023-00456-1 36695999 PMC9974679

[B21] Basnet J. Eissa M. A. Cardozo L. Romero D. G. Rezq S. (2024). Impact of probiotics and prebiotics on gut microbiome and hormonal regulation. Gastrointest. Disord. (Basel). 6, 801–815. 10.3390/gidisord6040056 39649015 PMC11623347

[B22] Beddows C. A. Shi F. Horton A. L. Dalal S. Zhang P. Ling C. C. (2024). Pathogenic hypothalamic extracellular matrix promotes metabolic disease. Nature 633, 914–922. 10.1038/s41586-024-07922-y 39294371 PMC11424483

[B23] Bellefontaine N. Chachlaki K. Parkash J. Vanacker C. Colledge W. d'Anglemont de Tassigny X. (2014). Leptin-dependent neuronal NO signaling in the preoptic hypothalamus facilitates reproduction. J. Clin. Invest 124 (6), 2550–2559. 10.1172/JCI65928 24812663 PMC4089460

[B24] Bhattarai J. P. Kim S. H. Han S. K. Park M. J. (2010). Effects of human growth hormone on gonadotropin-releasing hormone neurons in mice. Korean J. Pediatr. 53, 845–851. 10.3345/kjp.2010.53.9.845 21189970 PMC3010034

[B25] Bianchi V. E. Bresciani E. Meanti R. Rizzi L. Omeljaniuk R. J. Torsello A. (2021). The role of androgens in women's health and wellbeing. Pharmacol. Res. 171, 105758. 10.1016/j.phrs.2021.105758 34242799

[B26] Bilenka B. Ben-Shlomo I. Cozacov C. Gold C. H. Zohar S. (1995). Fertility, miscarriage and pregnancy after vertical banded gastroplasty operation for morbid obesity. Acta Obstet. Gynecol. Scand. 74 (1), 42–44. 10.3109/00016349509009942 7856431

[B27] Biro F. M. Galvez M. P. Greenspan L. C. Succop P. A. Vangeepuram N. Pinney S. M. (2010). Pubertal assessment method and baseline characteristics in a mixed longitudinal study of girls. Pediatrics 126 (3), e583–e590. 10.1542/peds.2009-3079 20696727 PMC4460992

[B28] Bo T. Liu M. Tang L. Lv J. Wen J. Wang D. (2022). Effects of high-fat diet during childhood on precocious puberty and gut microbiota in mice. Front. Microbiol. 13, 930747. 10.3389/fmicb.2022.930747 35910597 PMC9329965

[B29] Borgonovi T. F. Virgolin L. B. Janzantti N. S. Casarotti S. N. Penna A. (2022). Fruit bioactive compounds: effect on lactic acid bacteria and on intestinal microbiota. Food Res. Int. 161, 111809. 10.1016/j.foodres.2022.111809 36192952

[B30] Bromfield J. J. Sheldon I. M. (2011). Lipopolysaccharide initiates inflammation in Bovine granulosa cells *via* the TLR4 pathway and perturbs oocyte meiotic progression *in vitro* . Endocrinology 152 (12), 5029–5040. 10.1210/en.2011-1124 21990308 PMC3428914

[B31] Brothers K. J. Wu S. DiVall S. A. Messmer M. R. Kahn C. R. Miller R. S. (2010). Rescue of obesity-induced infertility in female mice due to a pituitary-specific knockout of the insulin receptor. Cell Metab. 12, 295–305. 10.1016/j.cmet.2010.06.010 20816095 PMC2935812

[B32] Brüning J. C. Gautam D. Burks D. J. Gillette J. Schubert M. Orban P. C. (2000). Role of brain insulin receptor in control of body weight and reproduction. Science 289 (5487), 2122–2125. 10.1126/science.289.5487.2122 11000114

[B33] Bu T. Sun Z. Pan Y. Deng X. Yuan G. (2024). Glucagon-like Peptide-1: new regulator in lipid metabolism. Diabetes Metab. J. 48, 354–372. 10.4093/dmj.2023.0277 38650100 PMC11140404

[B34] Butler M. G. (2016). Single gene and syndromic causes of obesity: illustrative examples. Prog. Mol. Biol. Transl. Sci. 140, 1–45. 10.1016/bs.pmbts.2015.12.003 27288824 PMC7377403

[B35] Caldwell A. Edwards M. C. Desai R. Jimenez M. Gilchrist R. B. Handelsman D. J. (2017). Neuroendocrine androgen action is a key Extraovarian mediator in the development of polycystic ovary syndrome. Proc. Natl. Acad. Sci. U. S. A. 114 (16), E3334–E3343. 10.1073/pnas.1616467114 28320971 PMC5402450

[B36] Cao L. Shitara H. Horii T. Nagao Y. Imai H. Abe K. (2007). The mitochondrial bottleneck occurs without reduction of mtDNA content in female mouse germ cells. Nat. Genet. 39 (3), 386–390. 10.1038/ng1970 17293866

[B37] Cao L. Du C. Zhai X. Li J. Meng J. Shao Y. (2022). Codonopsis pilosula polysaccharide improved spleen deficiency in mice by modulating gut microbiota and energy related metabolisms. Front. Pharmacol. 13, 862763. 10.3389/fphar.2022.862763 35559259 PMC9086242

[B38] Ceccatelli S. Hulting A. L. Zhang X. Gustafsson L. Villar M. Hökfelt T. (1993). Nitric oxide synthase in the rat anterior pituitary gland and the role of nitric oxide in regulation of luteinizing hormone secretion. Proc. Natl. Acad. Sci. U. S. A. 90 (23), 11292–11296. 10.1073/pnas.90.23.11292 7504302 PMC47968

[B39] Chabrolle C. Tosca L. Dupont J. (2007). Regulation of adiponectin and its receptors in rat ovary by human chorionic gonadotrophin treatment and potential involvement of adiponectin in granulosa cell steroidogenesis. Reproduction 133, 719–731. 10.1530/REP-06-0244 17504916

[B40] Chambers E. S. Byrne C. S. Morrison D. J. Murphy K. G. Preston T. Tedford C. (2019). Dietary supplementation with inulin-propionate ester or inulin improves insulin sensitivity in adults with overweight and obesity with distinct effects on the gut microbiota, plasma metabolome and systemic inflammatory responses: a randomised cross-over trial. Gut 68 (8), 1430–1438. 10.1136/gutjnl-2019-318424 30971437 PMC6691855

[B41] Chao S. Lu J. Li L. Guo H. Y. Xu K. Wang N. (2024). Maternal obesity may disrupt offspring metabolism by inducing oocyte genome hyper-methylation *via* increased DNMTs. Elife 13. 10.7554/eLife.97507 39642055 PMC11623932

[B42] Chen K. L. Madak-Erdogan Z. (2016). Estrogen and microbiota crosstalk: should we pay attention. Trends Endocrinol. Metab. 27 (11), 752–755. 10.1016/j.tem.2016.08.001 27553057

[B43] Chen M. J. Chou C. H. Chen S. U. Yang W. S. Yang Y. S. Ho H. N. (2015). The effect of androgens on ovarian follicle maturation: dihydrotestosterone suppress FSH-stimulated granulosa cell proliferation by upregulating PPARγ-dependent PTEN expression. Sci. Rep. 5, 18319. 10.1038/srep18319 26674985 PMC4682139

[B44] Chen D. Yan Y. Chen D. Ran L. Mi J. Lu L. (2019). Modulating effects of polysaccharides from the fruits ofLycium barbarumon the immune response and gut microbiota in cyclophosphamide-treated mice. Food Funct. 10, 3671–3683. 10.1039/c9fo00638a 31168539

[B45] Chen R. Wu X. Qiu H. Yang B. Chen Y. Chen X. (2023). Obesity-induced inflammatory miR-133a mediates apoptosis of granulosa cells and causes abnormal folliculogenesis. Acta Biochim. Biophys. Sin. (Shanghai) 55 (8), 1234–1246. 10.3724/abbs.2023089 37337633 PMC10448043

[B46] Chen Y. Ma G. Gai Y. Yang Q. Liu X. de Avila J. M. (2024). AMPK suppression due to obesity drives oocyte mtDNA heteroplasmy *via* ATF5-POLG axis. Adv. Sci. (Weinh) 11 (20), e2307480. 10.1002/advs.202307480 38499990 PMC11132083

[B47] Cheng L. Shi H. Jin Y. Li X. Pan J. Lai Y. (2016). Adiponectin deficiency leads to female subfertility and ovarian dysfunctions in mice. Endocrinology 157, 4875–4887. 10.1210/en.2015-2080 27700136

[B48] Ciangura C. Coupaye M. Deruelle P. Gascoin G. Calabrese D. Cosson E. (2019). Clinical practice guidelines for childbearing female candidates for bariatric surgery, pregnancy, and post-partum management after bariatric surgery. Obes. Surg. 29 (11), 3722–3734. 10.1007/s11695-019-04093-y 31493139

[B49] Cirillo D. Rachiglio A. M. la Montagna R. Giordano A. Normanno N. (2008). Leptin signaling in breast cancer: an overview. J. Cell Biochem. 105 (4), 956–964. 10.1002/jcb.21911 18821585

[B50] Constantin S. Reynolds D. Oh A. Pizano K. Wray S. (2021). Nitric oxide resets kisspeptin-excited GnRH neurons *via* PIP2 replenishment. Proc. Natl. Acad. Sci. U. S. A. 118 (1), e2012339118. 10.1073/pnas.2012339118 33443156 PMC7817197

[B51] Coquoz A. Regli D. Stute P. (2022). Impact of progesterone on the gastrointestinal tract: a comprehensive literature review. Climacteric 25 (4), 337–361. 10.1080/13697137.2022.2033203 35253565

[B52] Coutinho E. A. Prescott M. Hessler S. Marshall C. J. Herbison A. E. Campbell R. E. (2020). Activation of a classic hunger circuit slows luteinizing hormone pulsatility. Neuroendocrinology 110 (7-8), 671–687. 10.1159/000504225 31630145

[B53] Cowley M. A. Smart J. L. Rubinstein M. Cerdán M. G. Diano S. Horvath T. L. (2001). Leptin activates anorexigenic POMC neurons through a neural network in the arcuate nucleus. Nature 411 (6836), 480–484. 10.1038/35078085 11373681

[B54] Cox A. J. West N. P. Cripps A. W. (2015). Obesity, inflammation, and the gut microbiota. Lancet Diabetes Endocrinol. 3 (3), 207–215. 10.1016/S2213-8587(14)70134-2 25066177

[B55] Day E. A. Ford R. J. Smith B. K. Mohammadi-Shemirani P. Morrow M. R. Gutgesell R. M. (2019). Metformin-induced increases in GDF15 are important for suppressing appetite and promoting weight loss. Nat. Metab. 1 (12), 1202–1208. 10.1038/s42255-019-0146-4 32694673

[B203] Di Pietro M. Pascuali N. Parborell F. Abramovich D. (2018). Ovarian angiogenesis in polycystic ovary syndrome. Reproduction 155(5), R199–R209. 10.1530/REP-17-0597 29386378

[B172] de Medeiros S. F. Rodgers R. J. Norman R. J. (2021). Adipocyte and steroidogenic cell cross-talk in polycystic ovary syndrome. Hum. Reprod. Update 27(4), 771–796. 10.1093/humupd/dmab004 33764457

[B56] Devesa J. Caicedo D. (2019). The role of growth hormone on ovarian functioning and ovarian angiogenesis. Front. Endocrinol. (Lausanne) 10, 450. 10.3389/fendo.2019.00450 31379735 PMC6646585

[B57] Diamanti-Kandarakis E. Dunaif A. (2012). Insulin resistance and the polycystic ovary syndrome revisited: an update on mechanisms and implications. Endocr. Rev. 33 (6), 981–1030. 10.1210/er.2011-1034 23065822 PMC5393155

[B58] Díaz-Hernández V. Montaño L. M. Caldelas I. Marmolejo-Valencia A. (2022). A high-fat and high-carbohydrate diet promotes reminiscent hallmarks of an aging ovary in the rabbit model. Biomedicines 10 (12), 3068. 10.3390/biomedicines10123068 36551824 PMC9776075

[B59] Ding T. Yan W. Zhou T. Shen W. Wang T. Li M. (2022). Endocrine disrupting chemicals impact on ovarian aging: evidence from epidemiological and experimental evidence. Environ. Pollut. 305, 119269. 10.1016/j.envpol.2022.119269 35405219

[B60] Divall S. A. Williams T. R. Carver S. E. Koch L. Brüning J. C. Kahn C. R. (2010). Divergent roles of growth factors in the GnRH regulation of puberty in mice. J. Clin. Invest 120 (8), 2900–2909. 10.1172/JCI41069 20628204 PMC2912185

[B61] Douglass J. D. Dorfman M. D. Fasnacht R. Shaffer L. D. Thaler J. P. (2017). Astrocyte IKKβ/NF-κB signaling is required for diet-induced obesity and hypothalamic inflammation. Mol. Metab. 6 (4), 366–373. 10.1016/j.molmet.2017.01.010 28377875 PMC5369266

[B62] Duffy D. M. Ko C. Jo M. Brannstrom M. Curry T. E. (2019). Ovulation: parallels with inflammatory processes. Endocr. Rev. 40 (2), 369–416. 10.1210/er.2018-00075 30496379 PMC6405411

[B63] Duncan W. C. Nio-Kobayashi J. (2013). Targeting angiogenesis in the pathological ovary. Reprod. Fertil. Dev. 25 (2), 362–371. 10.1071/RD12112 22951108

[B64] Egan O. K. Inglis M. A. Anderson G. M. (2017). Leptin signaling in AgRP neurons modulates puberty onset and adult fertility in mice. J. Neurosci. 37 (14), 3875–3886. 10.1523/JNEUROSCI.3138-16.2017 28275162 PMC6596709

[B65] Elías-López A. L. Vázquez-Mena O. Sferruzzi-Perri A. N. (2023). Mitochondrial dysfunction in the offspring of obese mothers and it's transmission through damaged oocyte mitochondria: integration of mechanisms. Biochim. Biophys. Acta Mol. Basis Dis. 1869 (7), 166802. 10.1016/j.bbadis.2023.166802 37414229

[B66] Elmaleh-Sachs A. Schwartz J. L. Bramante C. T. Nicklas J. M. Gudzune K. A. Jay M. (2023). Obesity management in adults: a review. JAMA 330 (20), 2000–2015. 10.1001/jama.2023.19897 38015216 PMC11325826

[B67] Erdos E. Divoux A. Sandor K. Halasz L. Smith S. R. Osborne T. F. (2022). Unique role for lncRNA HOTAIR in defining depot-specific gene expression patterns in human adipose-derived stem cells. Genes Dev. 36 (9-10), 566–581. 10.1101/gad.349393.122 35618313 PMC9186385

[B68] Ervin S. M. Li H. Lim L. Roberts L. R. Liang X. Mani S. (2019). Gut microbial β-glucuronidases reactivate estrogens as components of the estrobolome that reactivate estrogens. J. Biol. Chem. 294 (49), 18586–18599. 10.1074/jbc.RA119.010950 31636122 PMC6901331

[B69] Escobar-Morreale H. F. Botella-Carretero J. I. Alvarez-Blasco F. Sancho J. San Millán J. L. (2005). The polycystic ovary syndrome associated with morbid obesity may resolve after weight loss induced by bariatric surgery. J. Clin. Endocrinol. Metab. 90 (12), 6364–6369. 10.1210/jc.2005-1490 16189250

[B70] Farkas I. Vastagh C. Farkas E. Bálint F. Skrapits K. Hrabovszky E. (2016). Glucagon-like Peptide-1 excites firing and increases GABAergic miniature postsynaptic currents (mPSCs) in gonadotropin-releasing hormone (GnRH) neurons of the Male mice *via* activation of nitric oxide (NO) and suppression of endocannabinoid signaling pathways. Front. Cell Neurosci. 10, 214. 10.3389/fncel.2016.00214 27672360 PMC5018486

[B71] Feješ A. Belvončíková P. Porcel Sanchis D. Borbélyová V. Celec P. Džunková M. (2024). The effect of cross-sex fecal microbiota transplantation on metabolism and hormonal status in adult rats. Int. J. Mol. Sci. 25 (1), 601. 10.3390/ijms25010601 38203771 PMC10778742

[B72] Feng Y. Tang Z. Zhang W. (2023). The role of macrophages in polycystic ovarian syndrome and its typical pathological features: a narrative review. Biomed. Pharmacother. 167, 115470. 10.1016/j.biopha.2023.115470 37716116

[B73] Fernández-Fernández R. Tena-Sempere M. Navarro V. M. Barreiro M. L. Castellano J. M. Aguilar E. (2005). Effects of ghrelin upon gonadotropin-releasing hormone and gonadotropin secretion in adult female rats: *in vivo* and *in vitro* studies. Neuroendocrinology 82, 245–255. 10.1159/000092753 16721030

[B74] Franks S. Hardy K. (2018). Androgen action in the ovary. Front. Endocrinol. (Lausanne) 9, 452. 10.3389/fendo.2018.00452 30147675 PMC6097027

[B75] Frayling T. M. Timpson N. J. Weedon M. N. Zeggini E. Freathy R. M. Lindgren C. M. (2007). A common variant in the FTO gene is associated with body mass index and predisposes to childhood and adult obesity. Science 316 (5826), 889–894. 10.1126/science.1141634 17434869 PMC2646098

[B76] Frazao R. Dungan Lemko H. M. da Silva R. P. Ratra D. V. Lee C. E. Williams K. W. (2014). Estradiol modulates Kiss1 neuronal response to ghrelin. Am. J. Physiol. Endocrinol. Metab. 306, E606–E614. 10.1152/ajpendo.00211.2013 24473434 PMC3948981

[B77] Frisch R. E. (2000). Female fertility and the body fat connection.

[B78] Frost G. Sleeth M. L. Sahuri-Arisoylu M. Lizarbe B. Cerdan S. Brody L. (2014). The short-chain fatty acid acetate reduces appetite *via* a central homeostatic mechanism. Nat. Commun. 5, 3611. 10.1038/ncomms4611 24781306 PMC4015327

[B79] Fu Z. Han L. Zhang P. Mao H. Zhang H. Wang Y. (2020). Cistanche polysaccharides enhance echinacoside absorption *in vivo* and affect the gut microbiota. Int. J. Biol. Macromol. 149, 732–740. 10.1016/j.ijbiomac.2020.01.216 31987946

[B80] Gao Y. Li W. Huang X. Lyu Y. Yue C. (2024). Advances in gut microbiota-targeted therapeutics for metabolic syndrome. Microorganisms 12, 851. 10.3390/microorganisms12050851 38792681 PMC11123306

[B81] Garcia D. N. Saccon T. D. Pradiee J. Rincón J. A. A. Andrade K. R. S. Rovani M. T. (2019). Effect of caloric restriction and rapamycin on ovarian aging in mice. Geroscience 41 (4), 395–408. 10.1007/s11357-019-00087-x 31359237 PMC6815295

[B82] Ge L. Sadeghirad B. Ball G. da Costa B. R. Hitchcock C. L. Svendrovski A. (2020). Comparison of dietary macronutrient patterns of 14 popular named dietary programmes for weight and cardiovascular risk factor reduction in adults: systematic review and network meta-analysis of randomised trials. BMJ 369, m696. 10.1136/bmj.m696 32238384 PMC7190064

[B83] Geng L. Yang X. Sun J. Ran X. Zhou D. Ye M. (2025). Gut microbiota modulation by inulin improves metabolism and ovarian function in polycystic ovary syndrome. Adv. Sci. (Weinh) 12, e2412558. 10.1002/advs.202412558 40192074 PMC12120758

[B84] Ghafouri-Fard S. Taheri M. (2021). The expression profile and role of non-coding RNAs in obesity. Eur. J. Pharmacol. 892, 173809. 10.1016/j.ejphar.2020.173809 33345852

[B85] Gonzalez M. B. Robker R. L. Rose R. D. (2022). Obesity and oocyte quality: significant implications for ART and emerging mechanistic insights. Biol. Reprod. 106 (2), 338–350. 10.1093/biolre/ioab228 34918035

[B86] Greenhill C. (2015). Gut microbiota: firmicutes and bacteroidetes involved in insulin resistance by mediating levels of glucagon-like peptide 1. Nat. Rev. Endocrinol. 11 (5), 254. 10.1038/nrendo.2015.40 25781856

[B87] Guan Z. Li H. Liu R. Cai C. Liu Y. Li J. (2023). Artificial intelligence in diabetes management: advancements, opportunities, and challenges. Cell Rep. Med. 4, 101213. 10.1016/j.xcrm.2023.101213 37788667 PMC10591058

[B88] Guo Z. Yu Q. (2019). Role of mTOR signaling in female reproduction. Front. Endocrinol. (Lausanne) 10, 692. 10.3389/fendo.2019.00692 31649622 PMC6794368

[B89] Guzzardi M. A. La Rosa F. Iozzo P. (2023). Trust the gut: outcomes of gut microbiota transplant in metabolic and cognitive disorders. Neurosci. Biobehav Rev. 149, 105143. 10.1016/j.neubiorev.2023.105143 36990372

[B90] Handa R. J. Reid D. L. Resko J. A. (1986). Androgen receptors in brain and pituitary of female rats: cyclic changes and comparisons with the male. Biol. Reprod. 34 (2), 293–303. 10.1095/biolreprod34.2.293 3485449

[B91] Hatton G. B. Ran S. Tranah T. H. Shawcross D. L. (2020). Lessons learned from faecal microbiota transplantation in cirrhosis. Curr. Hepatol. Rep. 19, 159–167. 10.1007/s11901-020-00520-2

[B92] Hessler S. Liu X. Herbison A. E. (2020). Direct inhibition of arcuate kisspeptin neurones by neuropeptide Y in the male and female mouse. J. Neuroendocrinol. 32 (5), e12849. 10.1111/jne.12849 32337804

[B93] Hill J. W. Elias C. F. Fukuda M. Williams K. W. Berglund E. D. Holland W. L. (2010). Direct insulin and leptin action on pro-opiomelanocortin neurons is required for normal glucose homeostasis and fertility. Cell Metab. 11 (4), 286–297. 10.1016/j.cmet.2010.03.002 20374961 PMC2854520

[B94] Hou Y. J. Zhu C. C. Duan X. Liu H. L. Wang Q. Sun S. C. (2016). Both diet and gene mutation induced obesity affect oocyte quality in mice. Sci. Rep. 6, 18858. 10.1038/srep18858 26732298 PMC4702149

[B95] Hua D. Zhou Y. Lu Y. Zhao C. Qiu W. Chen J. (2020). Lipotoxicity impairs granulosa cell function through activated endoplasmic reticulum stress pathway. Reprod. Sci. 27 (1), 119–131. 10.1007/s43032-019-00014-7 32046379

[B96] Huang J. Chen P. Xiang Y. Liang Q. Wu T. Liu J. (2022). Gut microbiota dysbiosis-derived macrophage pyroptosis causes polycystic ovary syndrome *via* steroidogenesis disturbance and apoptosis of granulosa cells. Int. Immunopharmacol. 107, 108717. 10.1016/j.intimp.2022.108717 35334358

[B97] Huang X. Liu J. Peng G. Lu M. Zhou Z. Jiang N. (2024a). Gut hormone multi-agonists for the treatment of type 2 diabetes and obesity: advances and challenges. J. Endocrinol. 262, e230404. 10.1530/JOE-23-0404 38916409

[B98] Huang F. Deng Y. Zhou M. Tang R. Zhang P. Chen R. (2024b). Fecal microbiota transplantation from patients with polycystic ovary syndrome induces metabolic disorders and ovarian dysfunction in germ-free mice. BMC Microbiol. 24, 364. 10.1186/s12866-024-03513-z 39333864 PMC11437718

[B99] Hussain T. Murtaza G. Kalhoro D. H. Kalhoro M. S. Metwally E. Chughtai M. I. (2021). Relationship between gut microbiota and host-metabolism: emphasis on hormones related to reproductive function. Anim. Nutr. 7 (1), 1–10. 10.1016/j.aninu.2020.11.005 33997325 PMC8110851

[B100] Hwang I. Park Y. J. Kim Y. R. Kim Y. N. Ka S. Lee H. Y. (2015). Alteration of gut microbiota by vancomycin and bacitracin improves insulin resistance *via* glucagon-like peptide 1 in diet-induced obesity. FASEB J. 29 (6), 2397–2411. 10.1096/fj.14-265983 25713030

[B101] Ibrahim S. S. Ibrahim R. S. Arabi B. Brockmueller A. Shakibaei M. Büsselberg D. (2024). The effect of GLP-1R agonists on the medical triad of obesity, diabetes, and cancer. Cancer Metastasis Rev. 43, 1297–1314. 10.1007/s10555-024-10192-9 38801466 PMC11554930

[B102] Irusta G. Abramovich D. Parborell F. Tesone M. (2010). Direct survival role of vascular endothelial growth factor (VEGF) on rat ovarian follicular cells. Mol. Cell Endocrinol. 325 (1-2), 93–100. 10.1016/j.mce.2010.04.018 20417686

[B103] Isola J. Hense J. D. Osório C. Biswas S. Alberola-Ila J. Ocanas S. R. (2024). Reproductive ageing: inflammation, immune cells, and cellular senescence in the aging ovary. Reproduction 168 e230499. 10.1530/REP-23-0499 38744316 PMC11301429

[B104] Israel D. D. Sheffer-Babila S. de Luca C. Jo Y. H. Liu S. M. Xia Q. (2012). Effects of leptin and melanocortin signaling interactions on pubertal development and reproduction. Endocrinology 153 (5), 2408–2419. 10.1210/en.2011-1822 22408174 PMC3381095

[B105] Izquierdo A. G. Crujeiras A. B. Casanueva F. F. Carreira M. C. (2019). Leptin, obesity, and leptin resistance: where are we 25 years later. Nutrients 11 (11), 2704. 10.3390/nu11112704 31717265 PMC6893721

[B106] Izzi-Engbeaya C. Dhillo W. S. (2022). Gut hormones and reproduction. Ann. Endocrinol. Paris. 83 (4), 254–257. 10.1016/j.ando.2022.06.003 35750201

[B107] Jacobsen S. H. Olesen S. C. Dirksen C. Jørgensen N. B. Bojsen-Møller K. N. Kielgast U. (2012). Changes in gastrointestinal hormone responses, insulin sensitivity, and beta-cell function within 2 weeks after gastric bypass in non-diabetic subjects. Obes. Surg. 22 (7), 1084–1096. 10.1007/s11695-012-0621-4 22359255

[B108] Jacquier E. F. van de Wouw M. Nekrasov E. Contractor N. Kassis A. Marcu D. (2024). Local and systemic effects of bioactive food ingredients: is there a role for functional foods to prime the gut for resilience. Foods 13, 739. 10.3390/foods13050739 38472851 PMC10930422

[B109] Jain A. Polotsky A. J. Rochester D. Berga S. L. Loucks T. Zeitlian G. (2007). Pulsatile luteinizing hormone amplitude and progesterone metabolite excretion are reduced in obese women. J. Clin. Endocrinol. Metab. 92 (7), 2468–2473. 10.1210/jc.2006-2274 17440019

[B110] Jasoni C. L. Todman M. G. Han S. K. Herbison A. E. (2005). Expression of mRNAs encoding receptors that mediate stress signals in gonadotropin-releasing hormone neurons of the mouse. Neuroendocrinology 82 (5-6), 320–328. 10.1159/000093155 16721036

[B111] Jia G. Fu Y. Zhao X. Dai Q. Zheng G. Yang Y. (2011). N6-methyladenosine in nuclear RNA is a major substrate of the obesity-associated FTO. Nat. Chem. Biol. 7 (12), 885–887. 10.1038/nchembio.687 22002720 PMC3218240

[B112] Jiang Y. Gao X. Liu Y. Yan X. Shi H. Zhao R. (2024). Cellular atlases of ovarian microenvironment alterations by diet and genetically-induced obesity. Sci. China Life Sci. 67 (1), 51–66. 10.1007/s11427-023-2360-3 37721638

[B113] Joyce S. A. Clarke D. J. (2024). Microbial metabolites as modulators of host physiology. Adv. Microb. Physiol. 84, 83–133. 10.1016/bs.ampbs.2023.12.001 38821635

[B114] Juhász A. E. Stubnya M. P. Teutsch B. Gede N. Hegyi P. Nyirády P. (2024). Ranking the dietary interventions by their effectiveness in the management of polycystic ovary syndrome: a systematic review and network meta-analysis. Reprod. Health 21 (1), 28. 10.1186/s12978-024-01758-5 38388374 PMC10885527

[B115] Jungari M. Choudhary A. Gill N. K. (2023). Comprehensive management of polycystic ovary syndrome: effect of pharmacotherapy, lifestyle modification, and enhanced adherence counseling. Cureus 15 (2), e35415. 10.7759/cureus.35415 36994287 PMC10042521

[B116] Juul F. Chang V. W. Brar P. Parekh N. (2017). Birth weight, early life weight gain and age at menarche: a systematic review of longitudinal studies. Obes. Rev. 18 (11), 1272–1288. 10.1111/obr.12587 28872224

[B117] Kawai T. Autieri M. V. Scalia R. (2021). Adipose tissue inflammation and metabolic dysfunction in obesity. Am. J. Physiol. Cell Physiol. 320 (3), C375–C391. 10.1152/ajpcell.00379.2020 33356944 PMC8294624

[B118] Khan D. Ojo O. O. Woodward O. R. Lewis J. E. Sridhar A. Gribble F. M. (2022). Evidence for involvement of GIP and GLP-1 receptors and the gut-gonadal axis in regulating female reproductive function in mice. Biomolecules 12 (12), 1736. 10.3390/biom12121736 36551163 PMC9775379

[B119] Kiani A. K. Medori M. C. Bonetti G. Aquilanti B. Velluti V. Matera G. (2022). Modern vision of the mediterranean diet. J. Prev. Med. Hyg. 63 (2 Suppl. 3), E36–E43. 10.15167/2421-4248/jpmh2022.63.2S3.2745 36479477 PMC9710405

[B120] Kim M. S. Shim I. Fahed A. C. Do R. Park W. Y. Natarajan P. (2024a). Association of genetic risk, lifestyle, and their interaction with obesity and obesity-related morbidities. Cell Metab. 36 (7), 1494–1503.e3. 10.1016/j.cmet.2024.06.004 38959863 PMC12285577

[B121] Kim S. M. Sultana F. Sims S. Gimenez-Roig J. Laurencin V. Pallapati A. (2024b). FSH, bone, belly and brain. J. Endocrinol. 262 (1), e230377. 10.1530/JOE-23-0377 38579764 PMC12991055

[B122] King S. E. Skinner M. K. (2020). Epigenetic transgenerational inheritance of obesity susceptibility. Trends Endocrinol. Metab. 31 (7), 478–494. 10.1016/j.tem.2020.02.009 32521235 PMC8260009

[B123] Klenov V. E. Jungheim E. S. (2014). Obesity and reproductive function: a review of the evidence. Curr. Opin. Obstet. Gynecol. 26, 455–460. 10.1097/GCO.0000000000000113 25254319

[B124] Koh H. E. Cao C. Mittendorfer B. (2022). Insulin clearance in obesity and type 2 diabetes. Int. J. Mol. Sci. 23 (2), 596. 10.3390/ijms23020596 35054781 PMC8776220

[B125] Kopchick J. J. Berryman D. E. Puri V. Lee K. Y. Jorgensen J. (2020). The effects of growth hormone on adipose tissue: old observations, new mechanisms. Nat. Rev. Endocrinol. 16, 135–146. 10.1038/s41574-019-0280-9 31780780 PMC7180987

[B126] Kovacs P. Parlow A. F. Karkanias G. B. (2002). Effect of centrally administered insulin on gonadotropin-releasing hormone neuron activity and luteinizing hormone surge in the diabetic female rat. Neuroendocrinology 76 (6), 357–365. 10.1159/000067585 12566943

[B127] Ku S. Haque M. A. Jang M. J. Ahn J. Choe D. Jeon J. I. (2024). The role of bifidobacterium in longevity and the future of probiotics. Food Sci. Biotechnol. 33, 2097–2110. 10.1007/s10068-024-01631-y 39130652 PMC11315853

[B128] Kwon S. A. Cho Y. S. (2025). Identification of loci associated with women's reproductive traits and exploration of a shared genetic basis with obesity. Hum. Genomics 19, 58. 10.1186/s40246-025-00773-2 40394640 PMC12093848

[B129] Lagaly D. V. Aad P. Y. Grado-Ahuir J. A. Hulsey L. B. Spicer L. J. (2008). Role of adiponectin in regulating ovarian theca and granulosa cell function. Mol. Cell Endocrinol. 284, 38–45. 10.1016/j.mce.2008.01.007 18289773

[B130] Lake J. K. Power C. Cole T. J. (1997). Women's reproductive health: the role of body mass index in early and adult life. Int. J. Obes. Relat. Metab. Disord. 21 (6), 432–438. 10.1038/sj.ijo.0800424 9192225

[B131] Landry D. A. Yakubovich E. Cook D. P. Fasih S. Upham J. Vanderhyden B. C. (2022). Metformin prevents age-associated ovarian fibrosis by modulating the immune landscape in female mice. Sci. Adv. 8 (35), eabq1475. 10.1126/sciadv.abq1475 36054356 PMC10848964

[B132] LeBlanc E. S. Patnode C. D. Webber E. M. Redmond N. Rushkin M. O'Connor E. A. (2018). Behavioral and pharmacotherapy weight loss interventions to prevent obesity-related morbidity and mortality in adults: updated evidence report and systematic review for the US preventive services task force. JAMA 320 (11), 1172–1191. 10.1001/jama.2018.7777 30326501 PMC13151892

[B133] Lee B. H. Indran I. R. Tan H. M. Li Y. Zhang Z. Li J. (2016). A dietary medium-chain fatty acid, decanoic acid, inhibits recruitment of Nur77 to the HSD3B2 promoter *in vitro* and reverses endocrine and metabolic abnormalities in a rat model of polycystic ovary syndrome. Endocrinology 157 (1), 382–394. 10.1210/en.2015-1733 26465200

[B134] Lee C. Y. Li S. Li X. F. Stalker D. A. E. Cooke C. Shao B. (2019). Lipopolysaccharide reduces gonadotrophin-releasing hormone (GnRH) gene expression: role of RFamide-related peptide-3 and kisspeptin. Reprod. Fertil. Dev. 31 (6), 1134–1143. 10.1071/RD18277 30922440

[B135] Legro R. S. Arslanian S. A. Ehrmann D. A. Hoeger K. M. Murad M. H. Pasquali R. (2013). Diagnosis and treatment of polycystic ovary syndrome: an endocrine society clinical practice guideline. J. Clin. Endocrinol. Metab. 98 (12), 4565–4592. 10.1210/jc.2013-2350 24151290 PMC5399492

[B136] Legro R. S. Dodson W. C. Kris-Etherton P. M. Kunselman A. R. Stetter C. M. Williams N. I. (2015). Randomized controlled trial of preconception interventions in infertile women with polycystic ovary syndrome. J. Clin. Endocrinol. Metab. 100 (11), 4048–4058. 10.1210/jc.2015-2778 26401593 PMC4702450

[B137] Leshan R. L. Greenwald-Yarnell M. Patterson C. M. Gonzalez I. E. Myers M. G. Jr (2012). Leptin action through hypothalamic nitric oxide synthase-1-expressing neurons controls energy balance. Nat. Med. 18 (5), 820–823. 10.1038/nm.2724 22522563 PMC3531967

[B138] Ley R. E. Turnbaugh P. J. Klein S. Gordon J. I. (2006). Microbial ecology: human gut microbes associated with obesity. Nature 444 (7122), 1022–1023. 10.1038/4441022a 17183309

[B139] Li Y. Zheng Q. Sun D. Cui X. Chen S. Bulbul A. (2019a). Dehydroepiandrosterone stimulates inflammation and impairs ovarian functions of polycystic ovary syndrome. J. Cell Physiol. 234 (5), 7435–7447. 10.1002/jcp.27501 30580448

[B140] Li S. Qi Y. Chen L. Qu D. Li Z. Gao K. (2019b). Effects of Panax ginseng polysaccharides on the gut microbiota in mice with antibiotic-associated diarrhea. Int. J. Biol. Macromol. 124, 931–937. 10.1016/j.ijbiomac.2018.11.271 30503788

[B141] Li Y. Shen L. Huang C. Li X. Chen J. Li S. C. (2021). Altered nitric oxide induced by gut microbiota reveals the connection between central precocious puberty and obesity. Clin. Transl. Med. 11 (2), e299. 10.1002/ctm2.299 33634977 PMC7842634

[B142] Li M. Chang Q. Luo Y. Pan J. Hu Y. Liu B. (2024). The gut microbial composition in polycystic ovary syndrome with hyperandrogenemia and its association with steroid hormones. Front. Cell Dev. Biol. 12, 1384233. 10.3389/fcell.2024.1384233 38872933 PMC11169812

[B143] Liang Z. Di N. Li L. Yang D. (2021). Gut microbiota alterations reveal potential gut-brain axis changes in polycystic ovary syndrome. J. Endocrinol. Invest 44 (8), 1727–1737. 10.1007/s40618-020-01481-5 33387350

[B144] Lin K. Zhu L. Yang L. (2022). Gut and obesity/metabolic disease: focus on microbiota metabolites. MedComm 3, e171. 10.1002/mco2.171 36092861 PMC9437302

[B145] Linnerbauer M. Wheeler M. A. Quintana F. J. (2020). Astrocyte crosstalk in CNS inflammation. Neuron 108 (4), 608–622. 10.1016/j.neuron.2020.08.012 32898475 PMC7704785

[B146] Liu G. Guo J. Zhang X. Lu Y. Miao J. Xue H. (2021). Obesity is a risk factor for central precocious puberty: a case-control study. BMC Pediatr. 21 (1), 509. 10.1186/s12887-021-02936-1 34784914 PMC8594221

[B147] Liu K. He X. Huang J. Yu S. Cui M. Gao M. (2023a). Short-chain fatty acid-butyric acid ameliorates granulosa cells inflammation through regulating METTL3-mediated N6-methyladenosine modification of FOSL2 in polycystic ovarian syndrome. Clin. Epigenetics 15 (1), 86. 10.1186/s13148-023-01487-9 37179374 PMC10183145

[B148] Liu Z. Xiao T. Liu H. (2023b). Leptin signaling and its central role in energy homeostasis. Front. Neurosci. 17, 1238528. 10.3389/fnins.2023.1238528 38027481 PMC10644276

[B149] Liu C. Yuan Y. Guo M. Xin Z. Chen G. J. Ding N. (2024). Rising incidence of obesity-related cancers among younger adults in China: a population-based analysis (2007–2021). Med 5, 1402–1412.e2. 10.1016/j.medj.2024.07.012 39181132 PMC11560649

[B150] Lively S. Schlichter L. C. (2018). Microglia responses to pro-inflammatory stimuli (LPS, IFNγ+TNFα) and reprogramming by resolving cytokines (IL-4, IL-10). Front. Cell Neurosci. 12, 215. 10.3389/fncel.2018.00215 30087595 PMC6066613

[B151] Locke A. E. Kahali B. Berndt S. I. Justice A. E. Pers T. H. Day F. R. (2015). Genetic studies of body mass index yield new insights for obesity biology. Nature 518 (7538), 197–206. 10.1038/nature14177 25673413 PMC4382211

[B152] Lonardo M. S. Cacciapuoti N. Guida B. Di Lorenzo M. Chiurazzi M. Damiano S. (2024). Hypothalamic-ovarian axis and adiposity relationship in polycystic ovary syndrome: physiopathology and therapeutic options for the management of metabolic and inflammatory aspects. Curr. Obes. Rep. 13 (1), 51–70. 10.1007/s13679-023-00531-2 38172476 PMC10933167

[B153] Long X. Yang Q. Qian J. Yao H. Yan R. Cheng X. (2022). Obesity modulates cell-cell interactions during ovarian folliculogenesis. iScience 25 (1), 103627. 10.1016/j.isci.2021.103627 35005562 PMC8718989

[B154] Loos R. Yeo G. (2022). The genetics of obesity: from discovery to biology. Nat. Rev. Genet. 23 (2), 120–133. 10.1038/s41576-021-00414-z 34556834 PMC8459824

[B155] López M. Nogueiras R. (2023). Ghrelin. Curr. Biol. 33, R1133–R1135. 10.1016/j.cub.2023.09.009 37935121

[B156] Louis G. W. Greenwald-Yarnell M. Phillips R. Coolen L. M. Lehman M. N. Myers M. G. Jr (2011). Molecular mapping of the neural pathways linking leptin to the neuroendocrine reproductive axis. Endocrinology 152 (6), 2302–2310. 10.1210/en.2011-0096 21427219 PMC3100610

[B157] Lu N. Li M. Lei H. Jiang X. Tu W. Lu Y. (2017). Butyric acid regulates progesterone and estradiol secretion *via* cAMP signaling pathway in Porcine granulosa cells. J. Steroid Biochem. Mol. Biol. 172, 89–97. 10.1016/j.jsbmb.2017.06.004 28602959

[B158] Luo Y. Qiao X. Ma Y. Deng H. Xu C. C. Xu L. (2020). Disordered metabolism in mice lacking irisin. Sci. Rep. 10 (1), 17368. 10.1038/s41598-020-74588-7 33060792 PMC7567109

[B159] Luo Y. Qiao X. Ma Y. Deng H. Xu C. C. Xu L. (2021). Irisin deletion induces a decrease in growth and fertility in mice. Reprod. Biol. Endocrinol. 19 (1), 22. 10.1186/s12958-021-00702-7 33581723 PMC7881587

[B160] Luo M. Chen Y. Pan X. Chen H. Fan L. Wen Y. E. (2023). *E. coli Nissle 1917* ameliorates mitochondrial injury of granulosa cells in polycystic ovary syndrome through promoting gut immune factor IL-22 *via* gut microbiota and microbial metabolism. Front. Immunol. 14, 1137089. 10.3389/fimmu.2023.1137089 37275915 PMC10235540

[B161] Lyu Q. Xue W. Liu R. Ma Q. Kasaragod V. B. Sun S. (2024). A brain-to-gut signal controls intestinal fat absorption. Nature 634, 936–943. 10.1038/s41586-024-07929-5 39261733

[B162] Ma K. Yin K. Li J. Ma L. Zhou Q. Lu X. (2024). The hypothalamic epigenetic landscape in dietary obesity. Adv. Sci. (Weinh) 11 (9), e2306379. 10.1002/advs.202306379 38115764 PMC10916675

[B163] MacGregor K. Ellefsen S. Pillon N. J. Hammarström D. Krook A. (2025). Sex differences in skeletal muscle metabolism in exercise and type 2 diabetes mellitus. Nat. Rev. Endocrinol. 21, 166–179. 10.1038/s41574-024-01058-9 39604583

[B164] Machtinger R. Combelles C. M. Missmer S. A. Correia K. F. Fox J. H. Racowsky C. (2012). The association between severe obesity and characteristics of failed fertilized oocytes. Hum. Reprod. 27 (11), 3198–3207. 10.1093/humrep/des308 22968161

[B165] MacKay H. Gunasekara C. J. Yam K. Y. Srisai D. Yalamanchili H. K. Li Y. (2022). Sex-specific epigenetic development in the mouse hypothalamic arcuate nucleus pinpoints human genomic regions associated with body mass index. Sci. Adv. 8 (39), eabo3991. 10.1126/sciadv.abo3991 36170368 PMC9519050

[B166] Maggard M. A. Yermilov I. Li Z. Maglione M. Newberry S. Suttorp M. (2008). Pregnancy and fertility following bariatric surgery: a systematic review. JAMA 300 (19), 2286–2296. 10.1001/jama.2008.641 19017915

[B167] Manaserh I. H. Chikkamenahalli L. Ravi S. Dube P. R. Park J. J. Hill J. W. (2019). Ablating astrocyte insulin receptors leads to delayed puberty and hypogonadism in mice. PLoS Biol. 17, e3000189. 10.1371/journal.pbio.3000189 30893295 PMC6443191

[B168] Martinez Guevara D. Vidal Cañas S. Palacios I. Gómez A. Estrada M. Gallego J. (2024). Effectiveness of probiotics, prebiotics, and synbiotics in managing insulin resistance and hormonal imbalance in women with polycystic ovary syndrome (PCOS): a systematic review of randomized clinical trials. Nutrients 16, 3916. 10.3390/nu16223916 39599701 PMC11597640

[B169] Martínez-Moreno C. G. Calderón-Vallejo D. Harvey S. Arámburo C. Quintanar J. L. (2018). Growth hormone (GH) and gonadotropin-releasing hormone (GnRH) in the central nervous system: a potential neurological combinatory therapy. Int. J. Mol. Sci. 19, 375. 10.3390/ijms19020375 29373545 PMC5855597

[B170] McFee R. M. Romereim S. M. Snider A. P. Summers A. F. Pohlmeier W. E. Kurz S. G. (2021). A high-androgen microenvironment inhibits granulosa cell proliferation and alters cell identity. Mol. Cell Endocrinol. 531, 111288. 10.1016/j.mce.2021.111288 33905753 PMC13104108

[B171] Mechanick J. I. Apovian C. Brethauer S. Garvey W. T. Joffe A. M. Kim J. (2019). Clinical practice guidelines for the perioperative nutrition, metabolic, and nonsurgical support of patients undergoing bariatric procedures - 2019 update: cosponsored by American association of clinical endocrinologists/american college of endocrinology, the obesity society, american society for metabolic and bariatric surgery, obesity medicine association, and american society of anesthesiologists - executive summary. Endocr. Pract. 25 (12), 1346–1359. 10.4158/GL-2019-0406 31682518

[B173] Merhi Z. O. (2009). Impact of bariatric surgery on female reproduction. Fertil. Steril. 92 (5), 1501–1508. 10.1016/j.fertnstert.2009.06.046 19665703

[B174] Merhi Z. Buyuk E. Berger D. S. Zapantis A. Israel D. D. Chua S. Jr (2013). Leptin suppresses Anti-Mullerian hormone gene expression through the JAK2/STAT3 pathway in luteinized granulosa cells of women undergoing IVF. Hum. Reprod. 28 (6), 1661–1669. 10.1093/humrep/det072 23503941

[B175] Mikhael S. Punjala-Patel A. Gavrilova-Jordan L. (2019). Hypothalamic-pituitary-ovarian axis disorders impacting female fertility. Biomedicines 7 (1), 5. 10.3390/biomedicines7010005 30621143 PMC6466056

[B176] Milstein J. L. Ferris H. A. (2021). The brain as an insulin-sensitive metabolic organ. Mol. Metab. 52, 101234. 10.1016/j.molmet.2021.101234 33845179 PMC8513144

[B177] Molina-Vega M. Muñoz-Garach A. Damas-Fuentes M. Fernández-García J. C. Tinahones F. J. (2018). Secondary Male hypogonadism: a prevalent but overlooked comorbidity of obesity. Asian J. Androl. 20 (6), 531–538. 10.4103/aja.aja_44_18 29974886 PMC6219298

[B178] Mollah M. L. Yang H. S. Jeon S. Kim K. Cheon Y. P. (2021). Overaccumulation of fat caused rapid reproductive senescence but not loss of ovarian reserve in ob/ob mice. J. Endocr. Soc. 5 (1), bvaa168. 10.1210/jendso/bvaa168 33324862 PMC7722705

[B179] Moore A. M. Prescott M. Marshall C. J. Yip S. H. Campbell R. E. (2015). Enhancement of a robust arcuate GABAergic input to gonadotropin-releasing hormone neurons in a model of polycystic ovarian syndrome. Proc. Natl. Acad. Sci. U. S. A. 112 (2), 596–601. 10.1073/pnas.1415038112 25550522 PMC4299257

[B180] Morais L. H. Schreiber H. L. Mazmanian S. K. (2021). The gut microbiota-brain axis in behaviour and brain disorders. Nat. Rev. Microbiol. 19 (4), 241–255. 10.1038/s41579-020-00460-0 33093662

[B181] Morales A. J. Laughlin G. A. Bützow T. Maheshwari H. Baumann G. Yen S. S. (1996). Insulin, somatotropic, and luteinizing hormone axes in lean and obese women with polycystic ovary syndrome: common and distinct features. J. Clin. Endocrinol. Metab. 81 (8), 2854–2864. 10.1210/jcem.81.8.8768842 8768842

[B182] Mueller N. T. Differding M. K. Zhang M. Maruthur N. M. Juraschek S. P. Miller E. R. (2021). Metformin affects gut microbiome composition and function and circulating short-chain fatty acids: a randomized trial. Diabetes Care 44, 1462–1471. 10.2337/dc20-2257 34006565 PMC8323185

[B183] Murphy C. T. Hu P. J. (2013). Insulin/insulin-like growth factor signaling in *C. elegans* . WormBook, 1–43. 10.1895/wormbook.1.164.1 24395814 PMC4780952

[B184] Navarro V. M. (2020). Metabolic regulation of kisspeptin - the link between energy balance and reproduction. Nat. Rev. Endocrinol. 16 (8), 407–420. 10.1038/s41574-020-0363-7 32427949 PMC8852368

[B185] Nie X. Xie R. Tuo B. (2018). Effects of estrogen on the gastrointestinal tract. Dig. Dis. Sci. 63 (3), 583–596. 10.1007/s10620-018-4939-1 29387989

[B186] Nishiyama Y. Hasegawa T. Fujita S. Iwata N. Nagao S. Hosoya T. (2018). Incretins modulate progesterone biosynthesis by regulating bone morphogenetic protein activity in rat granulosa cells. J. Steroid Biochem. Mol. Biol. 178, 82–88. 10.1016/j.jsbmb.2017.11.004 29129645

[B187] Nohara K. Waraich R. S. Liu S. Ferron M. Waget A. Meyers M. S. (2013). Developmental androgen excess programs sympathetic tone and adipose tissue dysfunction and predisposes to a cardiometabolic syndrome in female mice. Am. J. Physiol. Endocrinol. Metab. 304 (12), E1321–E1330. 10.1152/ajpendo.00620.2012 23612996 PMC3680697

[B188] Nøhr M. K. Pedersen M. H. Gille A. Egerod K. L. Engelstoft M. S. Husted A. S. (2013). GPR41/FFAR3 and GPR43/FFAR2 as cosensors for short-chain fatty acids in enteroendocrine cells vs FFAR3 in enteric neurons and FFAR2 in enteric leukocytes. Endocrinology 154 (10), 3552–3564. 10.1210/en.2013-1142 23885020

[B189] Nonaka H. Ohue-Kitano R. Masujima Y. Igarashi M. Kimura I. (2022). Dietary medium-chain triglyceride decanoate affects glucose homeostasis through GPR84-Mediated GLP-1 secretion in mice. Front. Nutr. 9, 848450. 10.3389/fnut.2022.848450 35399667 PMC8987919

[B190] Nteeba J. Ganesan S. Keating A. F. (2014). Progressive obesity alters ovarian folliculogenesis with impacts on pro-inflammatory and steroidogenic signaling in female mice. Biol. Reprod. 91 (4), 86. 10.1095/biolreprod.114.121343 25143355 PMC4435031

[B191] Nylander M. Frøssing S. Clausen H. V. Kistorp C. Faber J. Skouby S. O. (2017). Effects of liraglutide on ovarian dysfunction in polycystic ovary syndrome: a randomized clinical trial. Reprod. Biomed. Online 35 (1), 121–127. 10.1016/j.rbmo.2017.03.023 28479118

[B192] Oliveras-Cañellas N. Castells-Nobau A. de la Vega-Correa L. Latorre-Luque J. Motger-Albertí A. Arnoriaga-Rodriguez M. (2023). Adipose tissue coregulates cognitive function. Sci. Adv. 9 (32), eadg4017. 10.1126/sciadv.adg4017 37566655 PMC10421051

[B193] Outeiriño-Iglesias V. Romaní-Pérez M. González-Matías L. C. Vigo E. Mallo F. (2015). GLP-1 increases preovulatory LH source and the number of mature follicles, As well As synchronizing the onset of puberty in female rats. Endocrinology 156 (11), 4226–4237. 10.1210/en.2014-1978 26252058

[B194] Padilla S. L. Qiu J. Nestor C. C. Zhang C. Smith A. W. Whiddon B. B. (2017). AgRP to Kiss1 neuron signaling links nutritional state and fertility. Proc. Natl. Acad. Sci. U. S. A. 114 (9), 2413–2418. 10.1073/pnas.1621065114 28196880 PMC5338482

[B195] Padmanabhan V. Veiga-Lopez A. Abbott D. H. Recabarren S. E. Herkimer C. (2010). Developmental programming: impact of prenatal testosterone excess and postnatal weight gain on insulin sensitivity index and transfer of traits to offspring of overweight females. Endocrinology 151 (2), 595–605. 10.1210/en.2009-1015 19966179 PMC2817622

[B196] Pan B. Li J. (2019). The art of oocyte meiotic arrest regulation. Reprod. Biol. Endocrinol. 17 (1), 8. 10.1186/s12958-018-0445-8 30611263 PMC6320606

[B197] Pan D. Wang K. Cao G. Fan K. Liu H. Li P. (2020). Inhibitory effect of central ghrelin on steroid synthesis affecting reproductive health in female mice. J. Steroid Biochem. Mol. Biol. 204, 105750. 10.1016/j.jsbmb.2020.105750 32920127

[B198] Paoli A. Mancin L. Giacona M. C. Bianco A. Caprio M. (2020). Effects of a ketogenic diet in overweight women with polycystic ovary syndrome. J. Transl. Med. 18 (1), 104. 10.1186/s12967-020-02277-0 32103756 PMC7045520

[B199] Park H. K. Ahima R. S. (2015). Physiology of leptin: energy homeostasis, neuroendocrine function and metabolism. Metabolism 64, 24–34. 10.1016/j.metabol.2014.08.004 25199978 PMC4267898

[B200] Parker J. O'Brien C. Hawrelak J. Gersh F. L. (2022). Polycystic ovary syndrome: an evolutionary adaptation to lifestyle and the environment. Int. J. Environ. Res. Public Health 19 (3), 1336. 10.3390/ijerph19031336 35162359 PMC8835454

[B201] Peng S. L. Wu Q. F. Xie Q. Tan J. Shu K. Y. (2021). PATL2 regulated the apoptosis of ovarian granulosa cells in patients with PCOS. Gynecol. Endocrinol. 37 (7), 629–634. 10.1080/09513590.2021.1928066 34008465

[B202] Pérez-Pérez A. Sánchez-Jiménez F. Vilariño-García T. Sánchez-Margalet V. (2020). Role of leptin in inflammation and *vice versa* . Int. J. Mol. Sci. 21, 5887. 10.3390/ijms21165887 32824322 PMC7460646

[B204] Practice Committee of the American Society for Reproductive MedicinePractice Committee of the American Society for Reproductive Medicine (2021). Obesity and reproduction: a committee opinion. Fertil. Steril. 116 (5), 1266–1285. 10.1016/j.fertnstert.2021.08.018 34583840

[B205] Preziosi P. Barrett-Connor E. Papoz L. Roger M. Saint-Paul M. Nahoul K. (1993). Interrelation between plasma sex hormone-binding globulin and plasma insulin in healthy adult women: the telecom study. J. Clin. Endocrinol. Metab. 76 (2), 283–287. 10.1210/jcem.76.2.8432770 8432770

[B206] Qi X. Yun C. Sun L. Xia J. Wu Q. Wang Y. (2019a). Gut microbiota-bile acid-interleukin-22 axis orchestrates polycystic ovary syndrome. Nat. Med. 25 (8), 1225–1233. 10.1038/s41591-019-0509-0 31332392 PMC7376369

[B207] Qi Y. Chen L. Gao K. Shao Z. Huo X. Hua M. (2019b). Effects of Schisandra chinensis polysaccharides on rats with antibiotic-associated diarrhea. Int. J. Biol. Macromol. 124, 627–634. 10.1016/j.ijbiomac.2018.11.250 30500495

[B208] Qiu X. Dowling A. R. Marino J. S. Faulkner L. D. Bryant B. Brüning J. C. (2013). Delayed puberty but normal fertility in mice with selective deletion of insulin receptors from Kiss1 cells. Endocrinology 154 (3), 1337–1348. 10.1210/en.2012-2056 23392256 PMC3578993

[B209] Rak A. Mellouk N. Froment P. Dupont J. (2017). Adiponectin and resistin: potential metabolic signals affecting hypothalamo-pituitary gonadal axis in females and males of different species. Reproduction 153, R215–R226. 10.1530/REP-17-0002 28330882

[B210] Recabarren S. E. Padmanabhan V. Codner E. Lobos A. Durán C. Vidal M. (2005). Postnatal developmental consequences of altered insulin sensitivity in female sheep treated prenatally with testosterone. Am. J. Physiol. Endocrinol. Metab. 289 (5), E801–E806. 10.1152/ajpendo.00107.2005 16215166

[B211] Reynier P. May-Panloup P. Chrétien M. F. Morgan C. J. Jean M. Savagner F. (2001). Mitochondrial DNA content affects the fertilizability of human oocytes. Mol. Hum. Reprod. 7 (5), 425–429. 10.1093/molehr/7.5.425 11331664

[B212] Rich-Edwards J. W. Goldman M. B. Willett W. C. Hunter D. J. Stampfer M. J. Colditz G. A. (1994). Adolescent body mass index and infertility caused by ovulatory disorder. Am. J. Obstet. Gynecol. 171 (1), 171–177. 10.1016/0002-9378(94)90465-0 8030695

[B213] Rønnekleiv O. K. Qiu J. Kelly M. J. (2022). Hypothalamic kisspeptin neurons and the control of homeostasis. Endocrinology 163 (2), bqab253. 10.1210/endocr/bqab253 34953135 PMC8758343

[B214] Różańska-Walędziak A. Bartnik P. Kacperczyk-Bartnik J. Czajkowski K. Walędziak M. (2020). The impact of bariatric surgery on menstrual Abnormalities-a cross-sectional study. Obes. Surg. 30 (11), 4505–4509. 10.1007/s11695-020-04840-6 32661954 PMC7524851

[B215] Rubio-Ruiz M. E. Peredo-Escárcega A. E. Cano-Martínez A. Guarner-Lans V. (2015). An evolutionary perspective of nutrition and inflammation as mechanisms of cardiovascular disease. Int. J. Evol. Biol. 2015, 179791. 10.1155/2015/179791 26693381 PMC4677015

[B216] Ruebel M. L. Cotter M. Sims C. R. Moutos D. M. Badger T. M. Cleves M. A. (2017). Obesity modulates inflammation and lipid metabolism oocyte gene expression: a single-cell transcriptome perspective. J. Clin. Endocrinol. Metab. 102 (6), 2029–2038. 10.1210/jc.2016-3524 28323970 PMC5470765

[B217] Sakib M. N. Best J. R. Hall P. A. (2023). Bidirectional associations between adiposity and cognitive function and mediation by brain morphology in the ABCD study. JAMA Netw. Open 6 (2), e2255631. 10.1001/jamanetworkopen.2022.55631 36795417 PMC9936350

[B218] Salilew-Wondim D. Wang Q. Tesfaye D. Schellander K. Hoelker M. Hossain M. M. (2015). Polycystic ovarian syndrome is accompanied by repression of gene signatures associated with biosynthesis and metabolism of steroids, cholesterol and lipids. J. Ovarian Res. 8, 24. 10.1186/s13048-015-0151-5 25887459 PMC4414284

[B219] Saltiel A. R. (2021). Insulin signaling in health and disease. J. Clin. Invest 131, e142241. 10.1172/JCI142241 33393497 PMC7773347

[B220] Salum K. Assis I. Kopke Ú. A. Palhinha L. Abreu G. D. M. Gouvêa L. W. (2025). FTO rs17817449 variant increases the risk of severe obesity in a Brazilian cohort: a case-control study. Diabetes Metab. Syndr. Obes. 18, 283–303. 10.2147/DMSO.S451401 39906696 PMC11792641

[B221] Samuel B. S. Shaito A. Motoike T. Rey F. E. Backhed F. Manchester J. K. (2008). Effects of the gut microbiota on host adiposity are modulated by the short-chain fatty-acid binding G protein-coupled receptor, Gpr41. Proc. Natl. Acad. Sci. U. S. A. 105 (43), 16767–16772. 10.1073/pnas.0808567105 18931303 PMC2569967

[B222] Sansbury B. E. Hill B. G. (2014). Regulation of obesity and insulin resistance by nitric oxide. Free Radic. Biol. Med. 73, 383–399. 10.1016/j.freeradbiomed.2014.05.016 24878261 PMC4112002

[B223] Santoro N. Lasley B. McConnell D. Allsworth J. Crawford S. Gold E. B. (2004). Body size and ethnicity are associated with menstrual cycle alterations in women in the early menopausal transition: the study of Women's health across the nation (SWAN) daily hormone study. J. Clin. Endocrinol. Metab. 89 (6), 2622–2631. 10.1210/jc.2003-031578 15181033

[B224] Sarchielli E. Comeglio P. Squecco R. Ballerini L. Mello T. Guarnieri G. (2017). Tumor necrosis Factor-α impairs kisspeptin signaling in human gonadotropin-releasing hormone primary neurons. J. Clin. Endocrinol. Metab. 102 (1), 46–56. 10.1210/jc.2016-2115 27736314 PMC5413096

[B225] Sasaki H. Kawamura K. Kawamura T. Odamaki T. Katsumata N. Xiao J. Z. (2019). Distinctive subpopulations of the intestinal microbiota are present in women with unexplained chronic anovulation. Reprod. Biomed. Online 38 (4), 570–578. 10.1016/j.rbmo.2018.12.026 30773302

[B226] Schanbacher B. D. Johnson M. P. Tindall D. J. (1987). Androgenic regulation of luteinizing hormone secretion: relationship to androgen binding in sheep pituitary. Biol. Reprod. 36 (2), 340–350. 10.1095/biolreprod36.2.340 3580456

[B227] Schenkelaars N. Rousian M. Hoek J. Schoenmakers S. Willemsen S. Steegers-Theunissen R. (2021). Preconceptional maternal weight loss and hypertensive disorders in pregnancy: a systematic review and meta-analysis. Eur. J. Clin. Nutr. 75 (12), 1684–1697. 10.1038/s41430-021-00902-9 33837274

[B228] Schon S. B. Cabre H. E. Redman L. M. (2024). The impact of obesity on reproductive health and metabolism in reproductive-age females. Fertil. Steril. 122 (2), 194–203. 10.1016/j.fertnstert.2024.04.036 38704081 PMC11527540

[B229] Schwartz M. W. Seeley R. J. Campfield L. A. Burn P. Baskin D. G. (1996). Identification of targets of leptin action in rat hypothalamus. J. Clin. Invest 98 (5), 1101–1106. 10.1172/JCI118891 8787671 PMC507530

[B230] Sewaybricker L. E. Huang A. Chandrasekaran S. Melhorn S. J. Schur E. A. (2023). The significance of hypothalamic inflammation and gliosis for the pathogenesis of obesity in humans. Endocr. Rev. 44 (2), 281–296. 10.1210/endrev/bnac023 36251886 PMC10216879

[B231] Shao H. Xu C. Wang H. Lu N. Gu H. Zhang C. (2025). Dissecting the genetic determinants and biological associations between body mass index and female reproductive disorders based on genome-wide association study. Reprod. Biol. Endocrinol. 23, 71. 10.1186/s12958-025-01406-y 40369625 PMC12076840

[B232] Shen H. Xu X. Li X. (2021). Berberine exerts a protective effect on rats with polycystic ovary syndrome by inhibiting the inflammatory response and cell apoptosis. Reproductive Biol. Endocrinol. 19 (1), 3. 10.1186/s12958-020-00684-y 33407557 PMC7789273

[B233] Shen L. Liu J. Luo A. Wang S. (2023). The stromal microenvironment and ovarian aging: mechanisms and therapeutic opportunities. J. Ovarian Res. 16 (1), 237. 10.1186/s13048-023-01300-4 38093329 PMC10717903

[B234] Sher A. Rahman M. A. (2000). Enterohepatic recycling of estrogen and its relevance with female fertility. Arch. Pharm. Res. 23 (5), 513–517. 10.1007/BF02976582 11059833

[B235] Sherman S. B. Sarsour N. Salehi M. Schroering A. Mell B. Joe B. (2018). Prenatal androgen exposure causes hypertension and gut microbiota dysbiosis. Gut Microbes 9 (5), 400–421. 10.1080/19490976.2018.1441664 29469650 PMC6219642

[B236] Silva M. Desroziers E. Hessler S. Prescott M. Coyle C. Herbison A. E. (2019). Activation of arcuate nucleus GABA neurons promotes luteinizing hormone secretion and reproductive dysfunction: implications for polycystic ovary syndrome. EBioMedicine 44, 582–596. 10.1016/j.ebiom.2019.05.065 31178425 PMC6606966

[B237] Singh A. K. Singh R. (2020). Pharmacotherapy in obesity: a systematic review and meta-analysis of randomized controlled trials of anti-obesity drugs. Expert Rev. Clin. Pharmacol. 13 (1), 53–64. 10.1080/17512433.2020.1698291 31770497

[B238] Singh D. Arumalla K. Aggarwal S. Singla V. Ganie A. Malhotra N. (2020). Impact of bariatric surgery on clinical, biochemical, and hormonal parameters in women with polycystic ovary syndrome (PCOS). Obes. Surg. 30 (6), 2294–2300. 10.1007/s11695-020-04487-3 32088855

[B239] Snider A. P. Wood J. R. (2019). Obesity induces ovarian inflammation and reduces oocyte quality. Reproduction 158 (3), R79–R90. 10.1530/REP-18-0583 30999278

[B240] Speakman J. R. (2013). Evolutionary perspectives on the obesity epidemic: adaptive, maladaptive, and neutral viewpoints. Annu. Rev. Nutr. 33, 289–317. 10.1146/annurev-nutr-071811-150711 23862645

[B241] Speakman J. R. Elmquist J. K. (2022). Obesity: an evolutionary context. Life Metab. 1 (1), 10–24. 10.1093/lifemeta/loac002 36394061 PMC9642988

[B243] Stephens T. W. Basinski M. Bristow P. K. Bue-Valleskey J. M. Burgett S. G. Craft L. (1995). The role of neuropeptide Y in the antiobesity action of the obese gene product. Nature 377 (6549), 530–532. 10.1038/377530a0 7566151

[B244] Straub L. G. Scherer P. E. (2019). Metabolic messengers: adiponectin. Nat. Metab. 1, 334–339. 10.1038/s42255-019-0041-z 32661510 PMC7357716

[B245] Sundbom M. Holdstock C. Engström B. E. Karlsson F. A. (2007). Early changes in ghrelin following Roux-en-Y gastric bypass: influence of vagal nerve functionality. Obes. Surg. 17 (3), 304–310. 10.1007/s11695-007-9056-8 17546836

[B246] Takahashi Y. Morales Valencia M. Yu Y. Ouchi Y. Takahashi K. Shokhirev M. N. (2023). Transgenerational inheritance of acquired epigenetic signatures at CpG islands in mice. Cell 186 (4), 715–731.e19. 10.1016/j.cell.2022.12.047 36754048

[B247] Tanaka T. Nagatani S. Bucholtz D. C. Ohkura S. Tsukamura H. Maeda K. (2000). Central action of insulin regulates pulsatile luteinizing hormone secretion in the diabetic sheep model. Biol. Reprod. 62 (5), 1256–1261. 10.1095/biolreprod62.5.1256 10775174

[B248] Tatone C. Amicarelli F. Carbone M. C. Monteleone P. Caserta D. Marci R. (2008). Cellular and molecular aspects of ovarian follicle ageing. Hum. Reprod. Update 14 (2), 131–142. 10.1093/humupd/dmm048 18239135

[B249] Taylor C. C. Terranova P. F. (1995). Lipopolysaccharide inhibits rat ovarian thecal-interstitial cell steroid secretion *in vitro* . Endocrinology 136 (12), 5527–5532. 10.1210/endo.136.12.7588304 7588304

[B250] Taylor A. E. McCourt B. Martin K. A. Anderson E. J. Adams J. M. Schoenfeld D. (1997). Determinants of abnormal gonadotropin secretion in clinically defined women with polycystic ovary syndrome. J. Clin. Endocrinol. Metab. 82 (7), 2248–2256. 10.1210/jcem.82.7.4105 9215302

[B251] Teede H. J. Misso M. L. Costello M. F. Dokras A. Laven J. Moran L. (2018). Recommendations from the international evidence-based guideline for the assessment and management of polycystic ovary syndrome. Hum. Reprod. 33 (9), 1602–1618. 10.1093/humrep/dey256 30052961 PMC6112576

[B252] Teede H. J. Tay C. T. Joham A. E. (2021). Polycystic ovary syndrome: an intrinsic risk factor for diabetes compounded by obesity. Fertil. Steril. 115 (6), 1449–1450. 10.1016/j.fertnstert.2021.03.024 33865568

[B253] Tidblad A. (2022). The history, physiology and treatment safety of growth hormone. Acta Paediatr. 111, 215–224. 10.1111/apa.15948 34028879

[B254] Tolhurst G. Heffron H. Lam Y. S. Parker H. E. Habib A. M. Diakogiannaki E. (2012). Short-chain fatty acids stimulate glucagon-like peptide-1 secretion *via* the G-protein-coupled receptor FFAR2. Diabetes 61 (2), 364–371. 10.2337/db11-1019 22190648 PMC3266401

[B255] Trabert B. Weiss N. S. Rudra C. B. Scholes D. Holt V. L. (2011). A case-control investigation of adenomyosis: impact of control group selection on risk factor strength. Womens Health Issues 21 (2), 160–164. 10.1016/j.whi.2010.09.005 21269840 PMC3052973

[B256] Tremellen K. Pearce K. (2012). Dysbiosis of gut microbiota (DOGMA)--a novel theory for the development of polycystic ovarian syndrome. Med. Hypotheses 79 (1), 104–112. 10.1016/j.mehy.2012.04.016 22543078

[B257] Tremellen K. Syedi N. Tan S. Pearce K. (2015). Metabolic endotoxaemia--a potential novel link between ovarian inflammation and impaired progesterone production. Gynecol. Endocrinol. 31 (4), 309–312. 10.3109/09513590.2014.994602 25539190

[B258] Tropea A. Tiberi F. Minici F. Orlando M. Gangale M. F. Romani F. (2007). Ghrelin affects the release of luteolytic and luteotropic factors in human luteal cells. J. Clin. Endocrinol. Metab. 92, 3239–3245. 10.1210/jc.2007-0180 17535999

[B259] Turnbaugh P. J. Hamady M. Yatsunenko T. Cantarel B. L. Duncan A. Ley R. E. (2009). A core gut microbiome in obese and lean twins. Nature 457 (7228), 480–484. 10.1038/nature07540 19043404 PMC2677729

[B260] Ubba V. Joseph S. Awe O. Jones D. Dsilva M. K. Feng M. (2023). Neuronal AR regulates glucose homeostasis and energy expenditure in lean female mice with androgen excess. Endocrinology 164 (11), bqad141. 10.1210/endocr/bqad141 37738624 PMC12102723

[B261] Uddandrao V. Brahma Naidu P. Chandrasekaran P. Saravanan G. (2024). Pathophysiology of obesity-related infertility and its prevention and treatment by potential phytotherapeutics. Int. J. Obes. (Lond) 48 (2), 147–165. 10.1038/s41366-023-01411-4 37963998

[B262] Uenoyama Y. Inoue N. Nakamura S. Tsukamura H. (2021). Kisspeptin neurons and estrogen-estrogen receptor α signaling: unraveling the mystery of steroid feedback system regulating mammalian reproduction. Int. J. Mol. Sci. 22 (17), 9229. 10.3390/ijms22179229 34502135 PMC8430864

[B263] Umehara T. Winstanley Y. E. Andreas E. Morimoto A. Williams E. J. Smith K. M. (2022). Female reproductive life span is extended by targeted removal of fibrotic collagen from the mouse ovary. Sci. Adv. 8 (24), eabn4564. 10.1126/sciadv.abn4564 35714185 PMC9205599

[B264] Ussher J. R. Drucker D. J. (2023). Glucagon-like peptide 1 receptor agonists: cardiovascular benefits and mechanisms of action. Nat. Rev. Cardiol. 20, 463–474. 10.1038/s41569-023-00849-3 36977782

[B265] Valdearcos M. Douglass J. D. Robblee M. M. Dorfman M. D. Stifler D. R. Bennett M. L. (2017). Microglial inflammatory signaling orchestrates the hypothalamic immune response to dietary excess and mediates obesity susceptibility. Cell Metab. 26 (1), 185–197.e3. 10.1016/j.cmet.2017.05.015 28683286 PMC5569901

[B242] van der Steeg J. W. Steures P. Eijkemans M. J. Habbema J. D. F. Hompes P. G. A. Burggraaff J. M. (2008). Obesity affects spontaneous pregnancy chances in subfertile, ovulatory women. Hum. Reprod. 23(2), 324–328. 10.1093/humrep/dem371 18077317

[B266] Vasse G. F. Nizamoglu M. Heijink I. H. Schlepütz M. van Rijn P. Thomas M. J. (2021). Macrophage-stroma interactions in fibrosis: biochemical, biophysical, and cellular perspectives. J. Pathol. 254 (4), 344–357. 10.1002/path.5632 33506963 PMC8252758

[B267] Venkatesh S. S. Ferreira T. Benonisdottir S. Rahmioglu N. Becker C. M. Granne I. (2022). Obesity and risk of female reproductive conditions: a Mendelian randomisation study. PLoS Med. 19 (2), e1003679. 10.1371/journal.pmed.1003679 35104295 PMC8806071

[B268] Vogt E. C. Real F. G. Husebye E. S. Björnsdottir S. Benediktsdottir B. Bertelsen R. J. (2022). Premature menopause and autoimmune primary ovarian insufficiency in two international multi-center cohorts. Endocr. Connect. 11 (5), e220024. 10.1530/EC-22-0024 35521804 PMC9175594

[B269] Walsh J. Olavarria-Ramirez L. Lach G. Boehme M. Dinan T. G. Cryan J. F. (2020). Impact of host and environmental factors on β-glucuronidase enzymatic activity: implications for gastrointestinal serotonin. Am. J. Physiol. Gastrointest. Liver Physiol. 318 (4), G816–G826. 10.1152/ajpgi.00026.2020 32146834

[B270] Wang B. Cheng K. K. (2018). Hypothalamic AMPK as a mediator of hormonal regulation of energy balance. Int. J. Mol. Sci. 19, 3552. 10.3390/ijms19113552 30423881 PMC6274700

[B271] Wang N. Luo L. L. Xu J. J. Xu M. Y. Zhang X. M. Zhou X. L. (2014). Obesity accelerates ovarian follicle development and follicle loss in rats. Metabolism 63 (1), 94–103. 10.1016/j.metabol.2013.09.001 24135502

[B272] Wang H. Y. Guo S. C. Peng Z. T. Wang C. Duan R. Dong T. T. X. (2019). Ophiopogon polysaccharide promotes the *in vitro* metabolism of ophiopogonins by human gut microbiota. Molecules 24 (16), 2886. 10.3390/molecules24162886 31398918 PMC6719028

[B273] Wang D. Weng Y. Zhang Y. Wang R. Wang T. Zhou J. (2020). Exposure to hyperandrogen drives ovarian dysfunction and fibrosis by activating the NLRP3 inflammasome in mice. Sci. Total Environ. 745, 141049. 10.1016/j.scitotenv.2020.141049 32758727

[B274] Wang Z. Nie K. Su H. Tang Y. Wang H. Xu X. (2021). Berberine improves ovulation and endometrial receptivity in polycystic ovary syndrome. Phytomedicine 91, 153654. 10.1016/j.phymed.2021.153654 34333328

[B275] Wang L. Xu H. Tan B. Yi Q. Liu H. Deng H. (2022). Gut microbiota and its derived SCFAs regulate the HPGA to reverse obesity-induced precocious puberty in female rats. Front. Endocrinol. (Lausanne) 13, 1051797. 10.3389/fendo.2022.1051797 36568086 PMC9782419

[B276] Wang M. Pugh S. M. Daboul J. Miller D. Xu Y. Hill J. W. (2024a). IGF-1 acts through Kiss1-expressing cells to influence metabolism and reproduction. bioRxiv, 2024.07.02.601722. 10.1101/2024.07.02.601722 39005405 PMC11244982

[B277] Wang R. Z. He Y. Deng Y. T. Wang H. F. Zhang Y. Feng J. F. (2024b). Body weight in neurological and psychiatric disorders: a large prospective cohort study. Nat. Ment. Health 2 (1), 41–51. 10.1038/s44220-023-00158-1

[B278] Wei Y. Tan H. Yang R. Yang F. Liu D. Huang B. (2023). Gut dysbiosis-derived β-glucuronidase promotes the development of endometriosis. Fertil. Steril. 120 (3 Pt 2), 682–694. 10.1016/j.fertnstert.2023.03.032 37178109

[B279] Weisberg S. P. McCann D. Desai M. Rosenbaum M. Leibel R. L. Ferrante A. W. Jr (2003). Obesity is associated with macrophage accumulation in adipose tissue. J. Clin. Invest 112 (12), 1796–1808. 10.1172/JCI19246 14679176 PMC296995

[B280] Wen J. Feng Y. Xue L. Yuan S. Chen Q. Luo A. (2024). High-fat diet-induced L-saccharopine accumulation inhibits estradiol synthesis and damages oocyte quality by disturbing mitochondrial homeostasis. Gut Microbes 16 (1), 2412381. 10.1080/19490976.2024.2412381 39410876 PMC11485700

[B281] WHO (2025). Obesity and overweight. Available online at: https://www.who.int/en/news-room/fact-sheets/detail/obesity-and-overweight.

[B282] Winter S. E. Bäumler A. J. (2023). Gut dysbiosis: ecological causes and causative effects on human disease. Proc. Natl. Acad. Sci. U. S. A. 120 (50), e2316579120. 10.1073/pnas.2316579120 38048456 PMC10722970

[B283] Wołodko K. Castillo-Fernandez J. Kelsey G. Galvão A. (2021). Revisiting the impact of local leptin signaling in folliculogenesis and oocyte maturation in Obese mothers. Int. J. Mol. Sci. 22, 4270. 10.3390/ijms22084270 33924072 PMC8074257

[B284] Wu S. Divall S. Nwaopara A. Radovick S. Wondisford F. Ko C. (2014). Obesity-induced infertility and hyperandrogenism are corrected by deletion of the insulin receptor in the ovarian theca cell. Diabetes 63, 1270–1282. 10.2337/db13-1514 24379345 PMC3964497

[B285] Wu L. L. Russell D. L. Wong S. L. Chen M. Tsai T. S. St John J. C. (2015). Mitochondrial dysfunction in oocytes of Obese mothers: transmission to offspring and reversal by pharmacological endoplasmic reticulum stress inhibitors. Development 142 (4), 681–691. 10.1242/dev.114850 25670793

[B286] Xie L. Zhang D. Ma H. He H. Xia Q. Shen W. (2019). The effect of berberine on reproduction and metabolism in women with polycystic ovary syndrome: a systematic review and meta-analysis of randomized control trials. Evid. Based Complement. Altern. Med. 2019, 7918631. 10.1155/2019/7918631 31915452 PMC6930782

[B287] Xiong Y. L. Liang X. Y. Yang X. Li Y. Wei L. N. (2011). Low-grade chronic inflammation in the peripheral blood and ovaries of women with polycystic ovarian syndrome. Eur. J. Obstet. Gynecol. Reprod. Biol. 159 (1), 148–150. 10.1016/j.ejogrb.2011.07.012 21908093

[B288] Xu L. Zhang Q. Dou X. Wang Y. Wang J. Zhou Y. (2022). Fecal microbiota transplantation from young donor mice improves ovarian function in aged mice. J. Genet. Genomics 49 (11), 1042–1052. 10.1016/j.jgg.2022.05.006 35654347

[B289] Xu B. Qin W. Chen Y. Tang Y. Zhou S. Huang J. (2023). Multi-omics analysis reveals gut microbiota-ovary axis contributed to the follicular development difference between meishan and landrace × yorkshire sows. J. Anim. Sci. Biotechnol. 14 (1), 68. 10.1186/s40104-023-00865-w 37122038 PMC10150527

[B290] Yang P. K. Chou C. H. Huang C. C. Wen W. F. Chen H. F. Shun C. T. (2021). Obesity alters ovarian folliculogenesis through disrupted angiogenesis from increased IL-10 production. Mol. Metab. 49, 101189. 10.1016/j.molmet.2021.101189 33592337 PMC7933796

[B291] Yang J. Song Y. Gaskins A. J. Li L. J. Huang Z. Eriksson J. G. (2023). Mediterranean diet and female reproductive health over lifespan: a systematic review and meta-analysis. Am. J. Obstet. Gynecol. 229 (6), 617–631. 10.1016/j.ajog.2023.05.030 37506751

[B292] Yang M. Deng H. Zhou S. Lu D. Shen X. Huang L. (2024). Irisin alleviated the reproductive endocrinal disorders of PCOS mice accompanied by changes in gut microbiota and metabolomic characteristics. Front. Microbiol. 15, 1373077. 10.3389/fmicb.2024.1373077 38846566 PMC11153696

[B293] Ye W. Xie T. Song Y. Zhou L. (2021). The role of androgen and its related signals in PCOS. J. Cell Mol. Med. 25 (4), 1825–1837. 10.1111/jcmm.16205 33369146 PMC7882969

[B294] Ye Y. Zhou C. C. Hu H. Q. Fukuzawa I. Zhang H. L. (2022). Underlying mechanisms of acupuncture therapy on polycystic ovary syndrome: evidences from animal and clinical studies. Front. Endocrinol. (Lausanne) 13, 1035929. 10.3389/fendo.2022.1035929 36353235 PMC9637827

[B295] Yong W. Wang J. Leng Y. Li L. Wang H. (2023). Role of obesity in female reproduction. Int. J. Med. Sci. 20, 366–375. 10.7150/ijms.80189 36860674 PMC9969507

[B296] Yun Y. Wei Z. Hunter N. (2019). Maternal obesity enhances oocyte chromosome abnormalities associated with aging. Chromosoma 128 (3), 413–421. 10.1007/s00412-019-00716-6 31286204

[B297] Yura S. Ogawa Y. Sagawa N. Masuzaki H. Itoh H. Ebihara K. (2000). Accelerated puberty and late-onset hypothalamic hypogonadism in female transgenic skinny mice overexpressing leptin. J. Clin. Invest 105 (6), 749–755. 10.1172/JCI8353 10727443 PMC377463

[B298] Zaadstra B. M. Seidell J. C. Van Noord P. A. te Velde E. R. Habbema J. D. Vrieswijk B. (1993). Fat and female fecundity: prospective study of effect of body fat distribution on conception rates. BMJ 306 (6876), 484–487. 10.1136/bmj.306.6876.484 8448457 PMC1676805

[B299] Zhang J. Sun Z. Jiang S. Bai X. Ma C. Peng Q. (2019). Probiotic Bifidobacterium lactis V9 regulates the secretion of sex hormones in polycystic ovary syndrome patients through the gut-brain axis. mSystems 4 (2), e00017-19. 10.1128/mSystems.00017-19 31020040 PMC6469956

[B300] Zhang Y. Wang H. Zhang L. Yuan Y. Yu D. (2020). Codonopsis lanceolata polysaccharide CLPS alleviates high fat/high sucrose diet-induced insulin resistance *via* anti-oxidative stress. Int. J. Biol. Macromol. 145, 944–949. 10.1016/j.ijbiomac.2019.09.185 31669275

[B301] Zhang X. Huangfu Z. Wang S. (2023a). Review of mendelian randomization studies on age at natural menopause. Front. Endocrinol. (Lausanne) 14, 1234324. 10.3389/fendo.2023.1234324 37766689 PMC10520463

[B302] Zhang H. Zheng L. Li C. Jing J. Li Z. Sun S. (2023b). Effects of gut microbiota on omega-3-mediated ovary and metabolic benefits in polycystic ovary syndrome mice. J. Ovarian Res. 16, 138. 10.1186/s13048-023-01227-w 37443082 PMC10347784

[B303] Zhao Y. K. Gao Y. N. Wang L. C. Wang J. Wang G. J. Wu H. L. (2023). Correlation between abnormal energy metabolism of ovarian granulosa cells and *in vitro* fertilization-embryo transfer outcomes in patients with polycystic ovary syndrome and obesity. J. Ovarian Res. 16 (1), 145. 10.1186/s13048-023-01204-3 37480140 PMC10362761

[B304] Zheng H. Liang X. Zhou H. Zhou T. Liu X. Duan J. (2023). Integrated gut microbiota and fecal metabolome analyses of the effect of Lycium barbarum polysaccharide on D-galactose-induced premature ovarian insufficiency. Food Funct. 14 (15), 7209–7221. 10.1039/d3fo01659e 37463025

[B305] Zhou B. Coorperative Meta-Analysis Group Of China Obesity Task Force (2002). Predictive values of body mass index and waist circumference to risk factors of related diseases in Chinese adult population. Zhonghua Liu Xing Bing Xue Za Zhi 23 (1), 5–10. 10.3760/j.issn:0254-6450.2002.01.003 12015100

[B306] Zhou F. Shi L. B. Zhang S. Y. (2017). Ovarian fibrosis: a phenomenon of concern. Chin. Med. J. Engl. 130 (3), 365–371. 10.4103/0366-6999.198931 28139522 PMC5308021

[B307] Zhou J. Lin L. Liu L. Wang J. Xia G. Wang C. (2023). The transcriptome reveals the molecular regulatory network of primordial follicle depletion in obese mice. Fertil. Steril. 120 (4), 899–910. 10.1016/j.fertnstert.2023.05.165 37247688

[B308] Zhu J. Zhou Y. Jin B. Shu J. (2023). Role of estrogen in the regulation of central and peripheral energy homeostasis: from a menopausal perspective. Ther. Adv. Endocrinol. Metab. 14, 20420188231199359. 10.1177/20420188231199359 37719789 PMC10504839

